# Supplementary material for: Site-selective protonation enables efficient carbon monoxide electroreduction to acetate
Source: Nat Commun. 2024 Jan 19;15:616. doi: 10.1038/s41467-024-44727-z (PMC10798983; doi:10.1038/s41467-024-44727-z)
Supplement: Supplementary file 1 — Supplementary Information [file 41467_2024_44727_MOESM1_ESM.pdf]

Supplementary information

Site-selective protonation enables efficient carbon monoxide electroreduction to acetate

Authors: Xinyue Wang[1,2,4], Yuanjun Chen[1,4], Feng Li[3,4], Rui Kai Miao[3,4], Jianan Erick Huang[1], Zilin Zhao[2], Xiao-Yan Li[1], Roham Dorakhan[1], Senlin Chu[2], Jinhong Wu[3], Sixing Zheng[2], Weiyan Ni[1], Dongha Kim[1], Sungjin Park[1], Yongxiang Liang[1], Adnan Ozden[3], Pengfei Ou[1], Yang Hou[2*], David Sinton[3*] and Edward H. Sargent[1*]

Affiliations:

[1]Department of Electrical and Computer Engineering, University of Toronto, Toronto, ON M5S 1A4 Canada.

[2]Key Laboratory of Biomass Chemical Engineering of Ministry of Education, College of Chemical and Biological Engineering, Zhejiang University, Hangzhou 310027, China.

[3]Department of Mechanical and Industrial Engineering, University of Toronto, Toronto, ON M5S 3G8, Canada.

[4]These authors contributed equally: Xinyue Wang, Yuanjun Chen, Feng Li, Rui Kai Miao

*Corresponding author. Email: ted.sargent@utoronto.ca (E.H.S), sinton@mie.utoronto.ca (D.S.), yhou@zju.edu.cn (Y.H.)

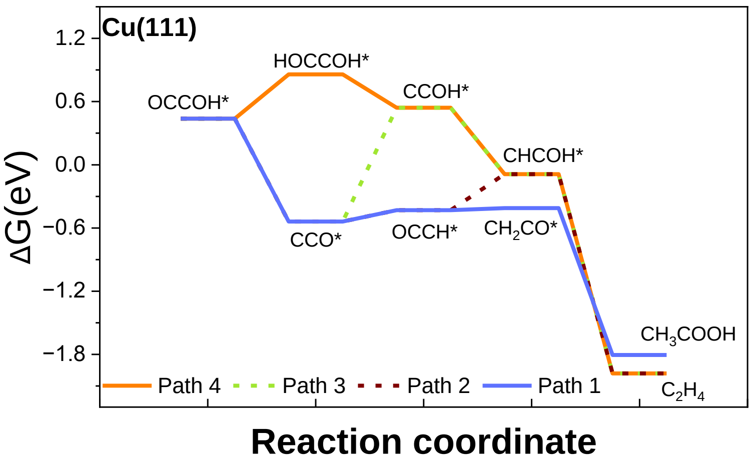

Supplementary Fig. 1 | Gibbs free energy profiles of various reaction pathways to acetate and ethylene on Cu(111) surface. The ethylene production via path 3 and 4 is not energetically favorable due to the high energy state of HOCCOH* and CCOH* intermediates, respectively.

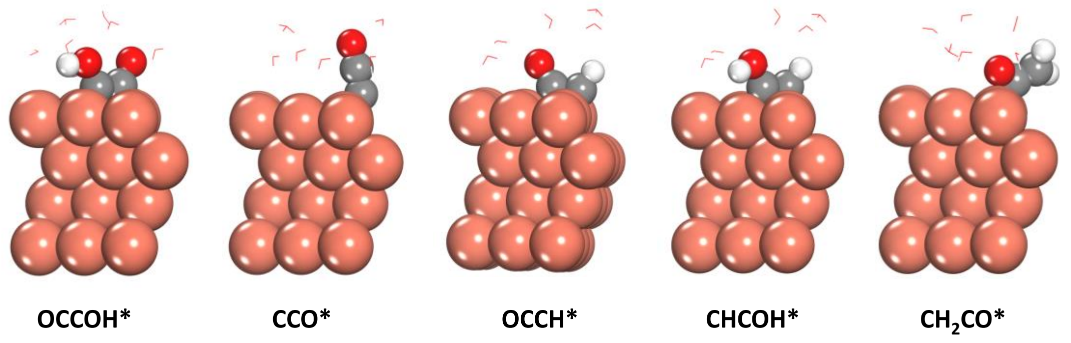

Supplementary Fig. 2 | Optimized geometries of various COR intermediates on Cu(111).

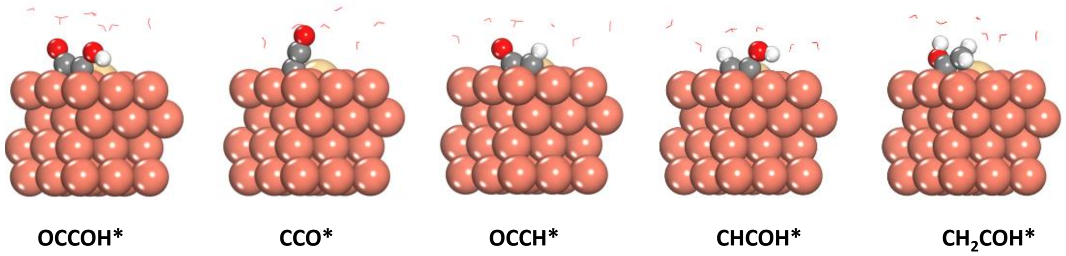

Supplementary Fig. 3 | Optimized geometries of various COR intermediates on Cd-Cu.

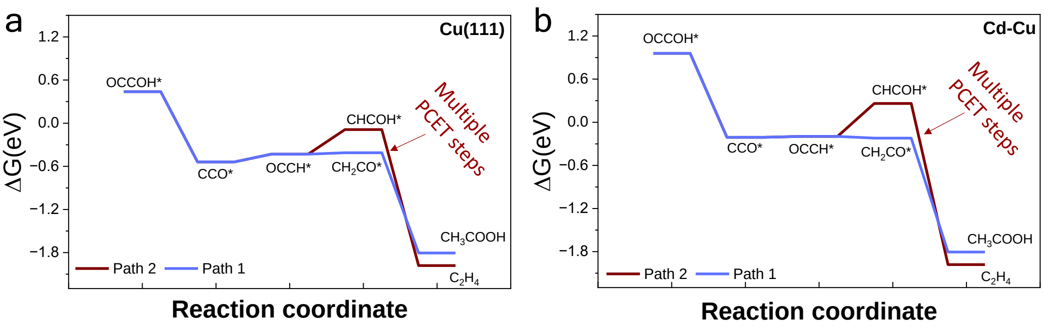

Supplementary Fig. 4 | Gibbs free energy profiles of COR to acetate and ethylene on (a) Cu(111) and (b) Cd-Cu surfaces.

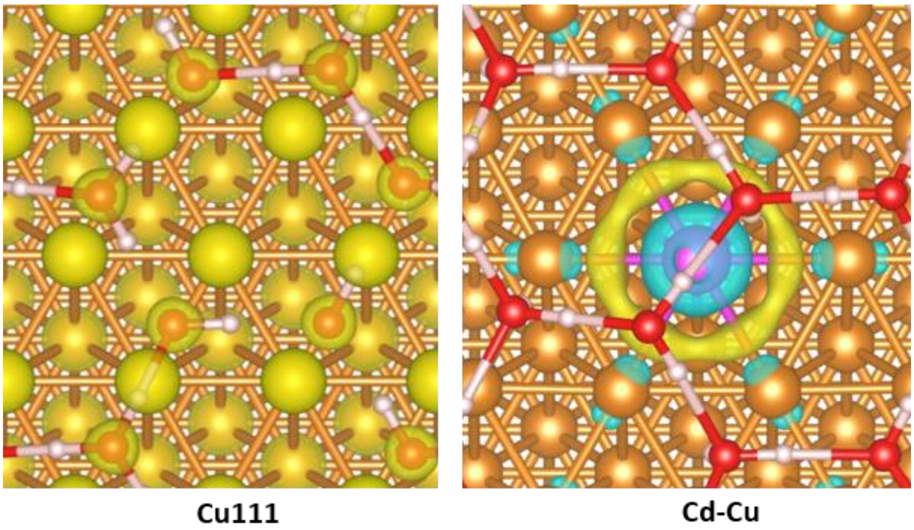

Supplementary Fig. 5 | Charge density difference on Cu surface with and without the doped Cd atom. Yellow and blue contours represent the isosurfaces of electronic charge accumulation and depletion, respectively, with an iso-surface value of 0.003 eÅ^{-3} implemented.

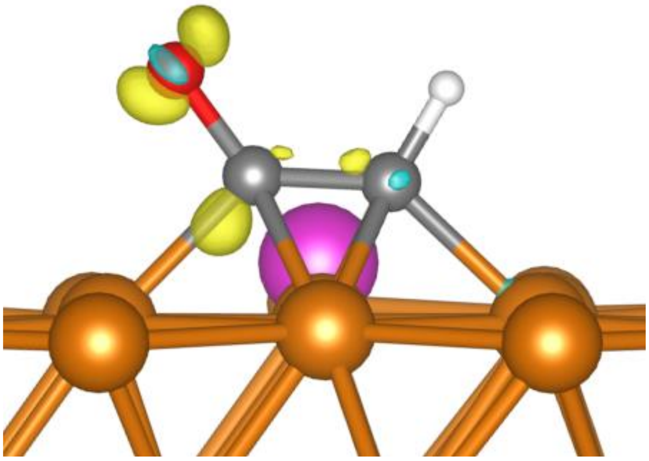

Supplementary Fig. 6 | Charge density difference of adsorbed OCCH* intermediate on the Cd-Cu surface. Yellow and blue contours represent the isosurfaces of electronic charge accumulation and depletion, respectively, with an iso-surface value of 0.03 eÅ^{-3} implemented.

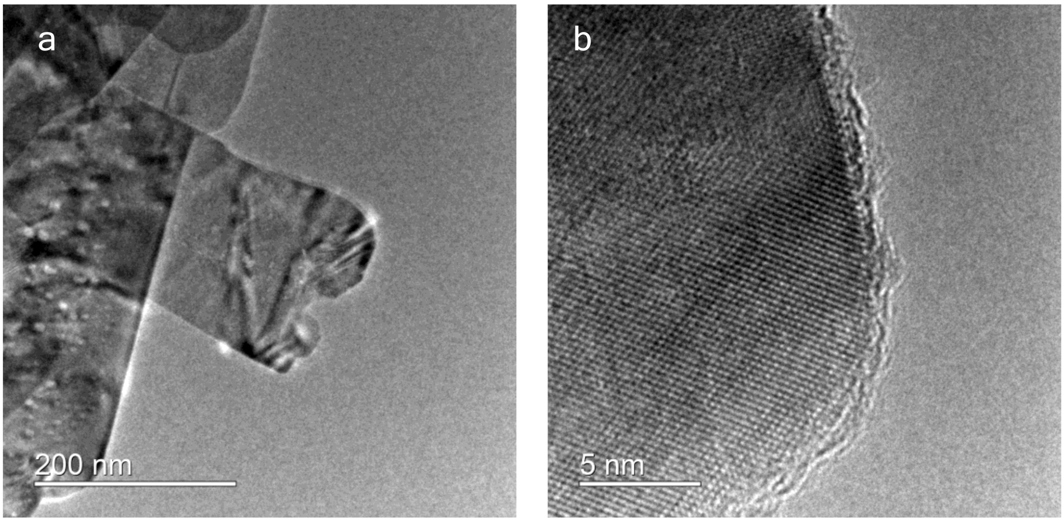

Supplementary Fig. 7 | Morphology characterization of the performed Cd-Cu catalyst. (a) TEM image and (b) HR-TEM image of the performed Cd-Cu catalyst.

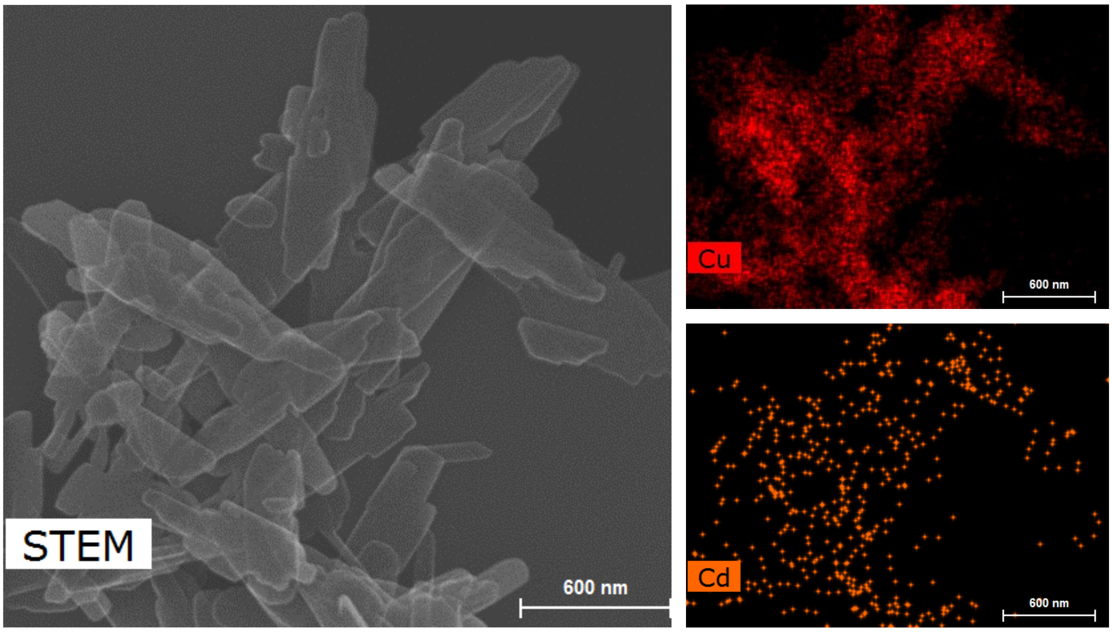

Supplementary Fig. 8 | STEM image (left) and the corresponding EDX mapping images (Cu and Cd) of Cd-Cu catalyst.

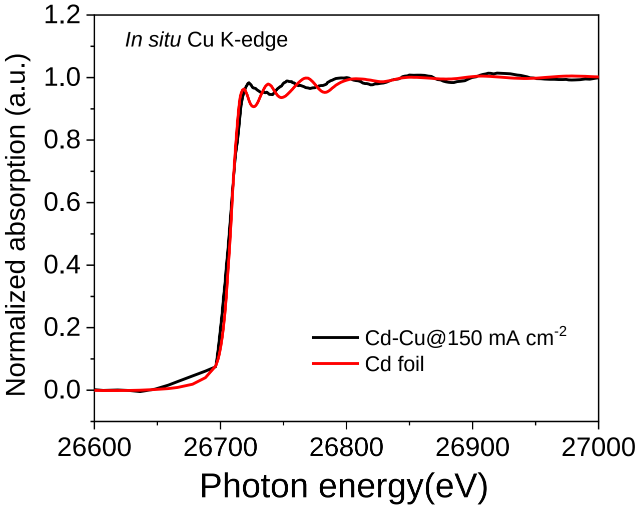

Supplementary Fig. 9 | *In-situ* Cd K-edge normalized XANES spectra under 150 mA cm^{-2}.

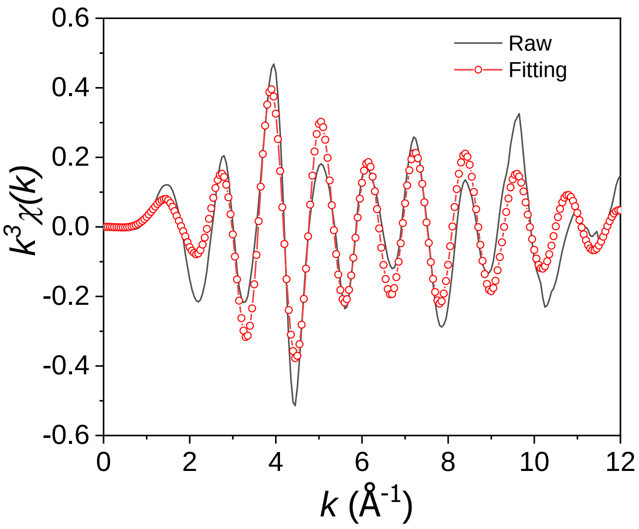

Supplementary Fig. 10 | *In-situ* Cd K-edge EXAFS k space fitting curves of the Cd-Cu catalysts under 150 mA cm^{-2}.

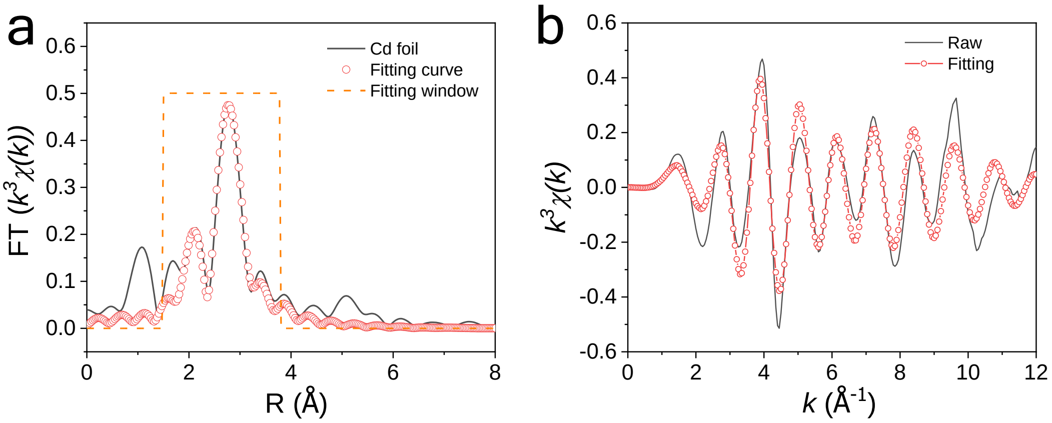

Supplementary Fig. 11 | Cd K-edge EXAFS in (a) R and (b) k space fitting curves of Cd foil.

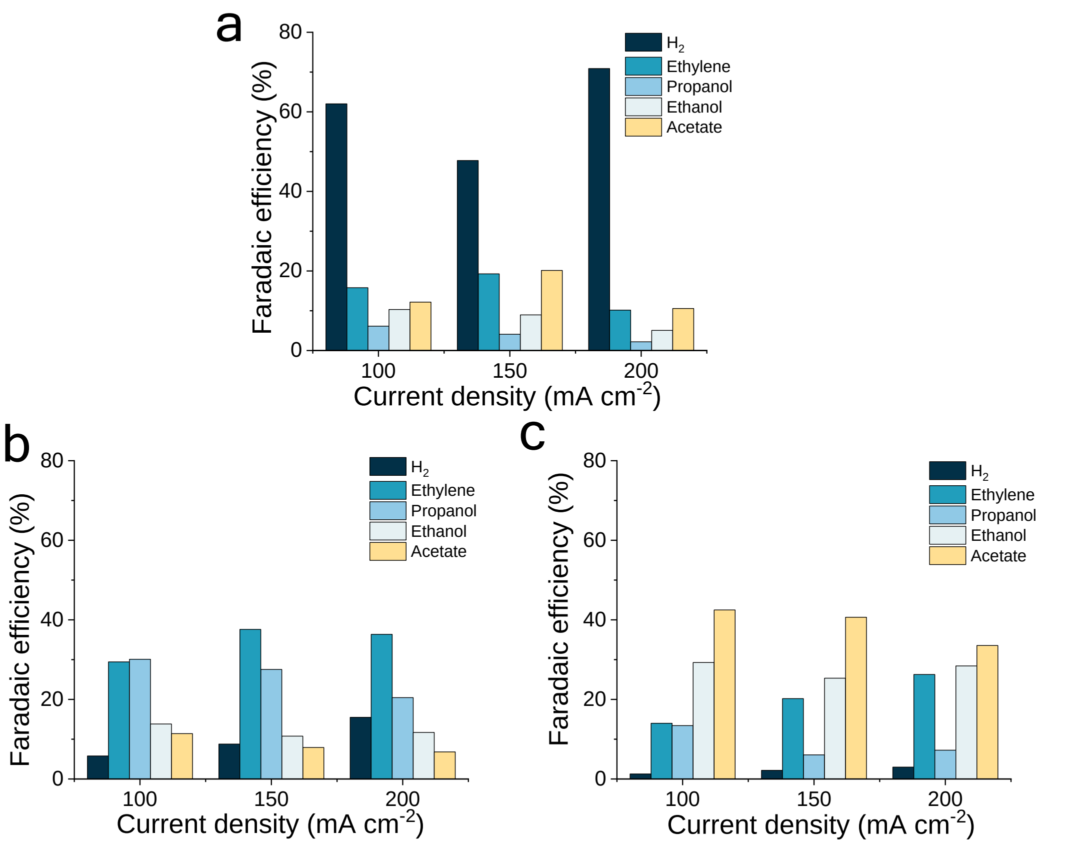

Supplementary Fig. 12 | Products distribution in COR on the Co-Cu (a), Pd-Cu (b) and Au-Cu (c) electrodes under different current densities.

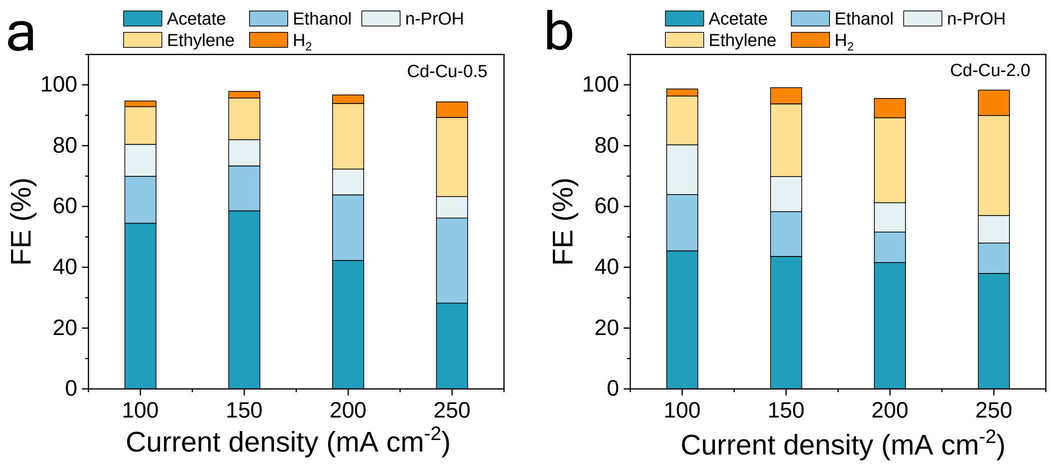

Supplementary Fig. 13 | COR performance of Cd-Cu catalysts with the different Cd loading. (a) The Cd dosages of 0.5 wt% and (b) 2 wt%.

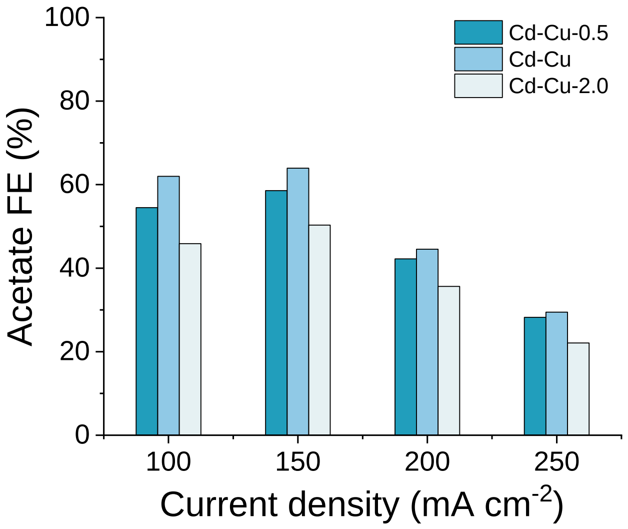

Supplementary Fig. 14 | The comparison of acetate FE on Cd-Cu catalysts with the different Cd loading.

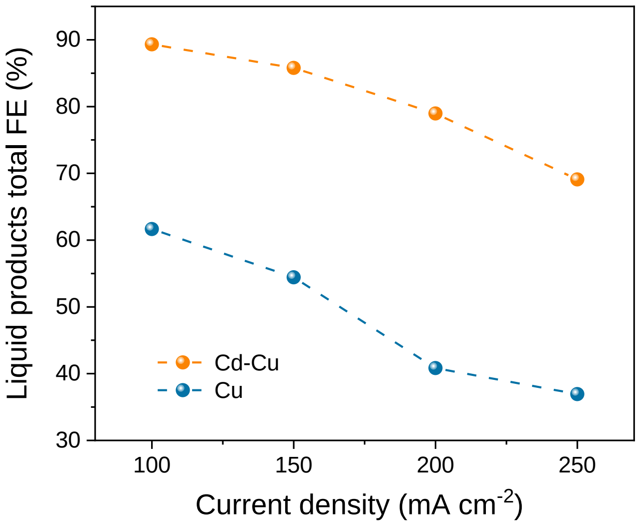

Supplementary Fig. 15 | Liquid products total FE on the Cd-Cu and Cu electrodes under different current densities.

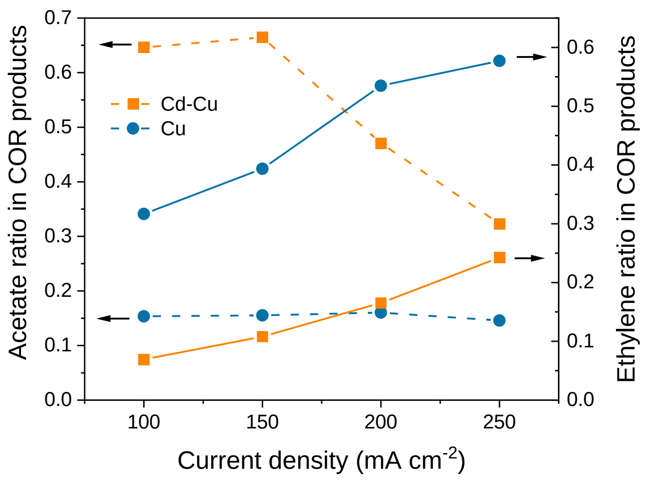

Supplementary Fig. 16 | Acetate and ethylene FE ratio in COR products on the Cd-Cu and Cu electrodes under different current densities.

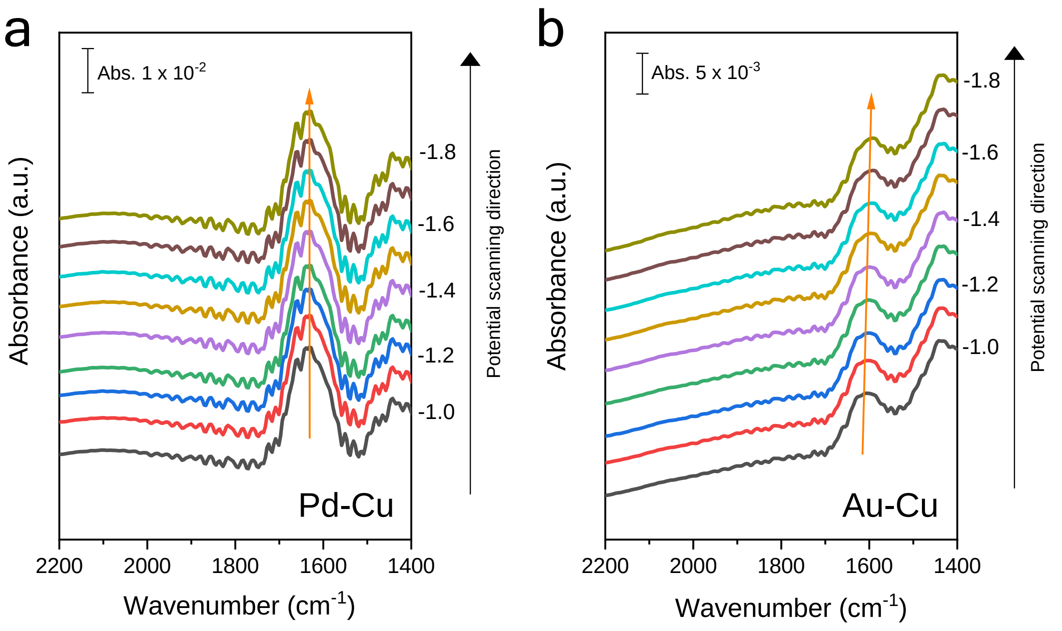

Supplementary Fig. 17 | *Operando* ATR-SEIRAS spectra of (a) the Pd-Cu electrode, and (b) the Au-Cu electrode. Spectra presented correspond to 64 coadded scans collected with an 8 cm^{-1} resolution. a.u., arbitrary units.

We selected Pd-Cu and Au-Cu in light of their weaker and stronger H affinities compared with pure Cu (Cd < Au < Cu < Pd). The Stark tuning rates of *OH on Pd-Cu and Au-Cu are 1 cm^{-1}/V and 18 cm^{-1}/V. This trend, where the Stark tuning rate increases as the H affinity decreases, correlates with the selectivity of acetate, is consistent with a picture wherein acetate formation is influenced by the tuning of H affinity.

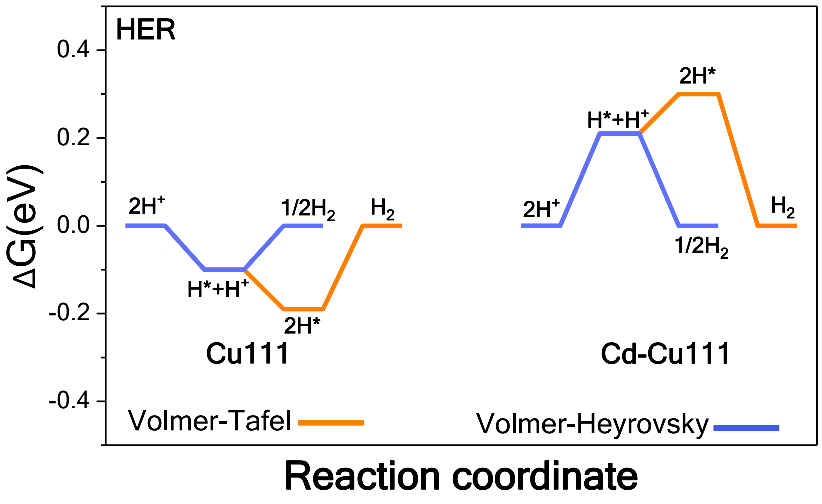

Supplementary Fig. 18 | Gibbs free energy profiles of HER on Cu(111) and Cd-Cu surfaces. DFT calculations indicate that the doping of Cd atom to Cu(111) surface alters the potential-determining step of HER in the Volmer-Tafel mechanism from the H_2 formation and desorption step ($2H^*$ -> H_2) to H^+ adsorption step ($2H^+$ -> H^*+H^+), which can be attributed to the much weaker H adsorption strength on Cd-Cu(111) than that on Cu(111). Cd-Cu(111) shows higher energy barriers in the potential-determining step of HER in both Volmer-Tafel and Volmer-Heyrovsky mechanisms (0.21 eV in both mechanisms on Cd-Cu(111), while 0.11 eV and 0.19 eV on Cu(111) for Volmer-Heyrovsky and Volmer-Tafel mechanism, respectively.

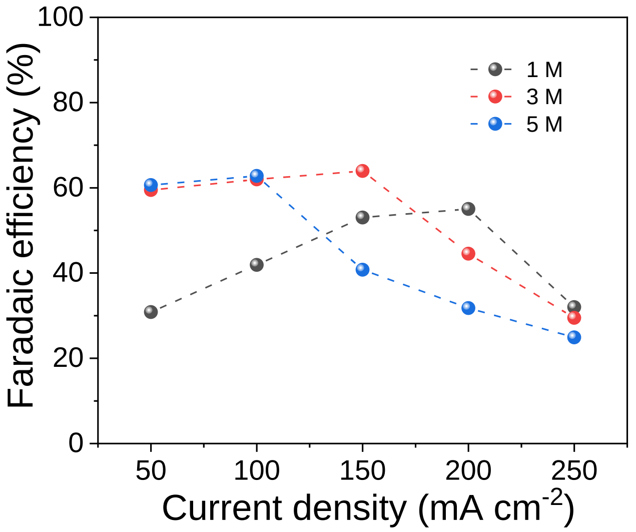

Supplementary Fig. 19 | Acetate FE on Cd-Cu electrode in various KOH concentrations.

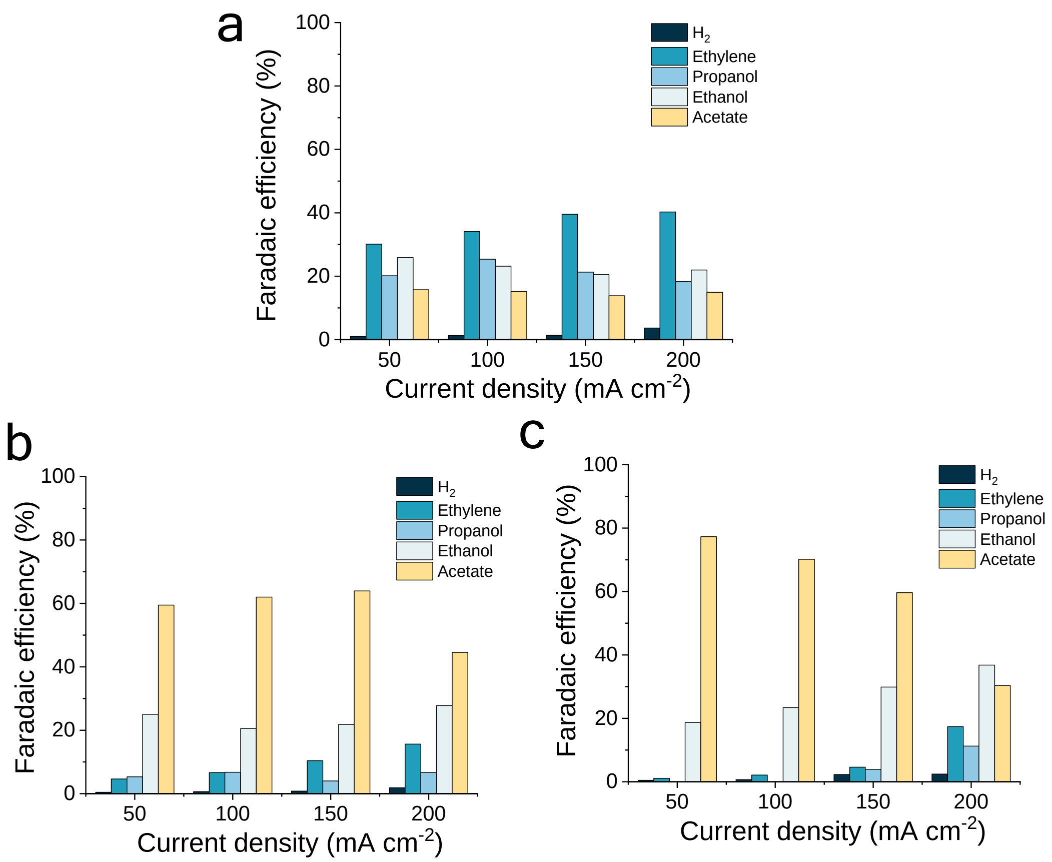

Supplementary Fig. 20 | Products distribution in COR on Cd-Cu electrode in various 3 M alkali metal cations electrolyte under different current densities. (a) 3 M NaOH, (b) 3 M KOH and (c) 3 M CsOH.

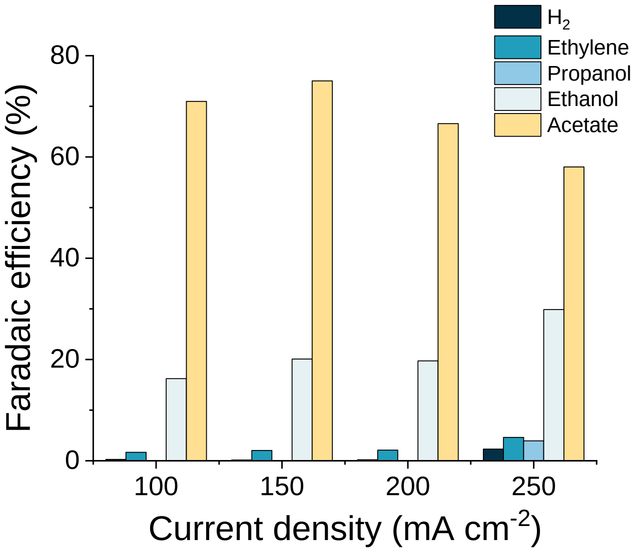

Supplementary Fig. 21 | Products distribution in COR on Cd-Cu electrode in 2 M KOH + 1 M CsOH electrolyte under different current densities.

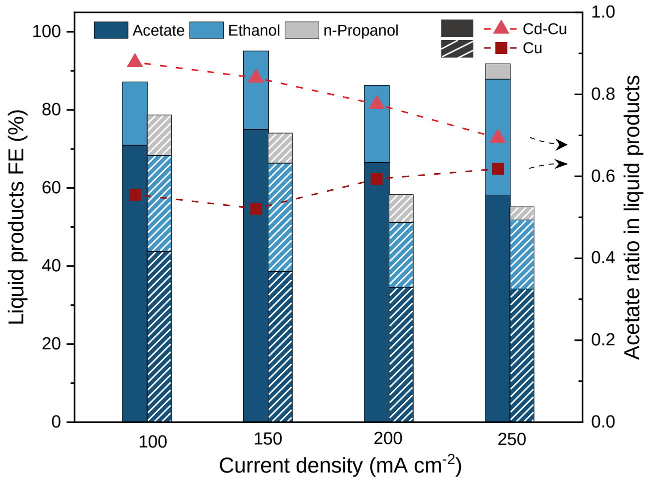

Supplementary Fig. 22 | Liquid products FE and acetate FE ratio in liquid products on the Cd-Cu and Cu electrodes in 2 M KOH + 1 M CsOH electrolyte under different current densities.

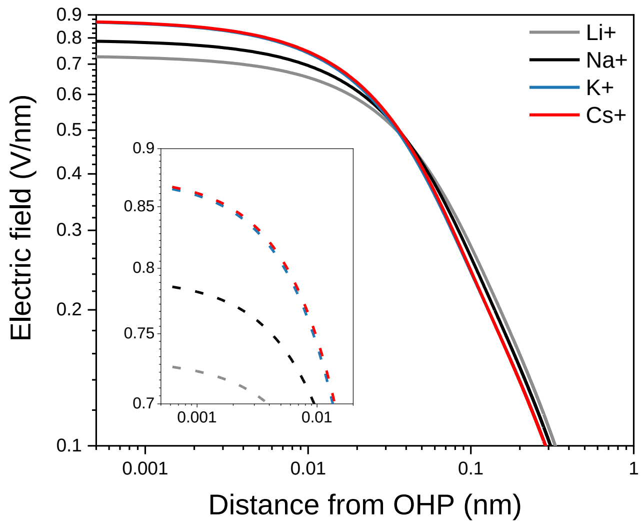

Supplementary Fig. 23 | Plot of electric filed near the electrode surface with different alkali metal cations.

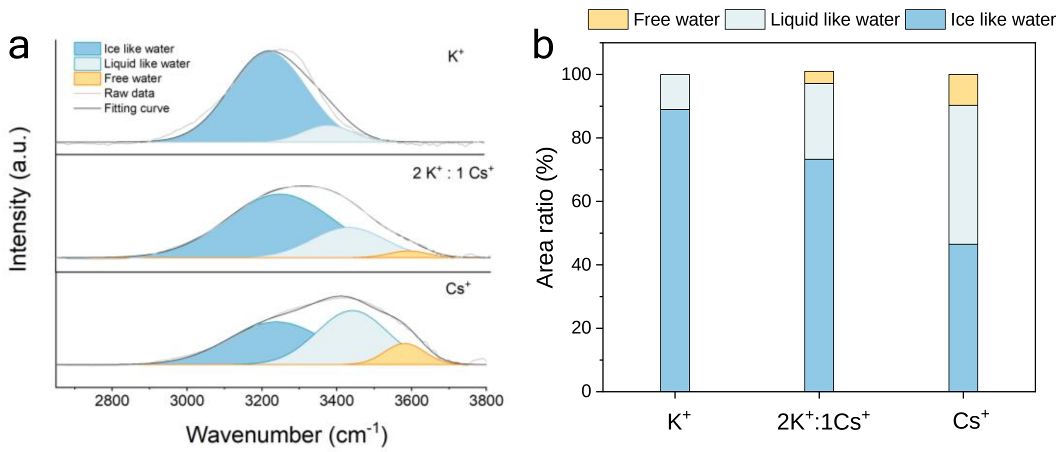

Supplementary Fig. 24 | Behavior of interfacial water on Cd-Cu catalysts. (a) *Operando* ATR–SEIRAS spectra (grey curves) of the interfacial water on Cd-Cu in different electrolytes with varying ratios of K^+ and Cs^+. These were fitted with three Gaussians (blue: ice like water; light blue: liquid like water; and yellow: free water, respectively). (b) The relationship between the area ratios of the three peaks at -1.7 V.

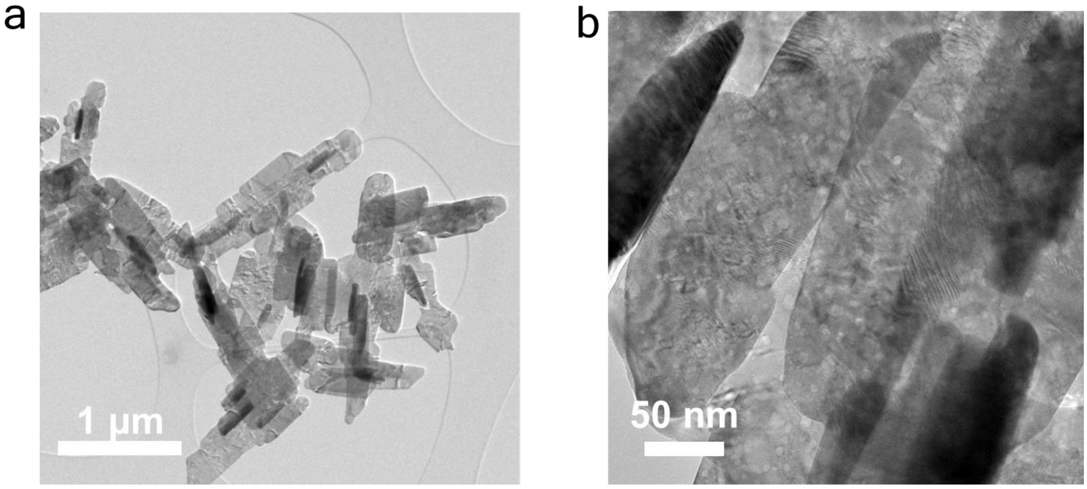

Supplementary Fig. 25 | Morphology characterization of the post-reaction Cd-Cu catalyst. (a) TEM image and (b) HR-TEM image of the used Cd-Cu catalyst after a 20 h stability test.

We collected the post-reaction Cd-Cu sample after stability test and performed structural characterizations. TEM images show the well-preserved nanoplates morphology and no Cd nanoparticles are observed.

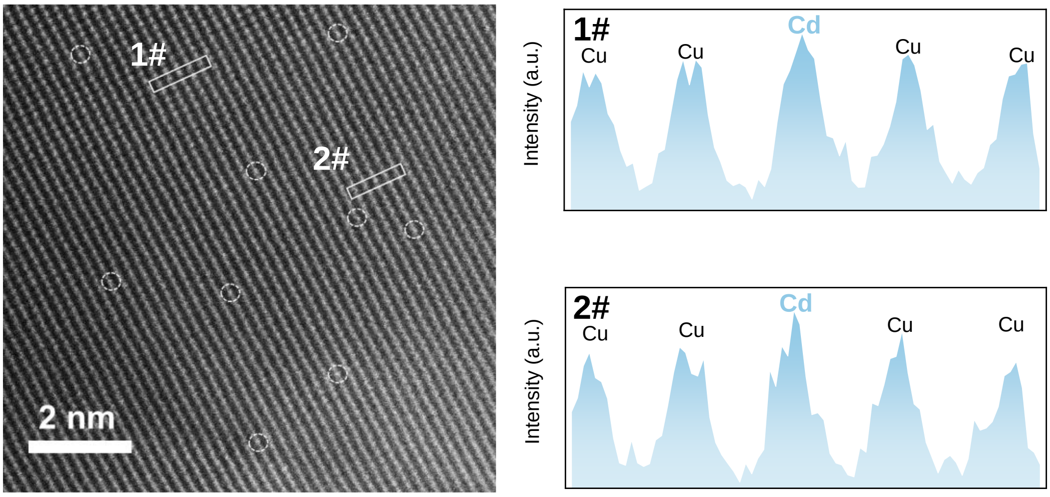

Supplementary Fig. 26 | The AC HAADF-STEM image of the used Cd-Cu catalyst after a 20 h stability test. The intensity profiles along the white solid lines.

The AC HAADF-STEM image suggests that no evidence of Cd agglomeration. The Cd single atoms are identified by the heightened intensity profiles of the areas marked in the AC HAADF-STEM image. The findings suggest that the structure and atomic dispersion of Cd are well-maintained, demonstrating a high stability of Cd-Cu.

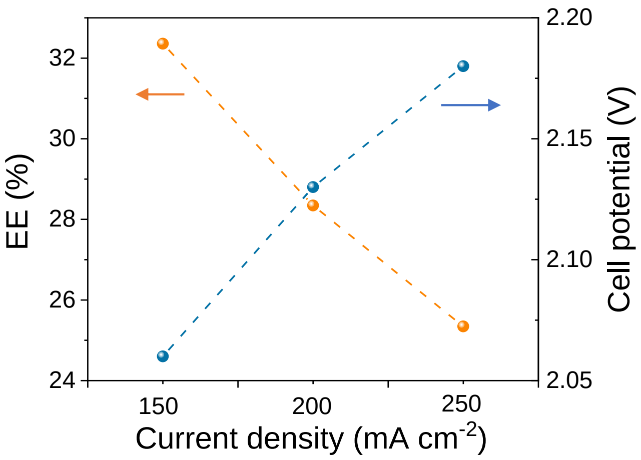

Supplementary Fig. 27 | Energy efficiency and cell potential of Cd-Cu electrode under high pressure of 8 bar in 2 M KOH + 1 M CsOH electrolyte.

Supplementary Table 1. Performance comparison of CO toward acetate among state-of-the-art electrocatalysts.

Catalyst	$FE_{Acetate}$	Energy efficiency	Ref
**Cd-Cu**	**86% (8 bar)**<br>**75%**	**32%**<br>**29%**	**This work**
Cu/Ag-DA	91% (10 atm)<br>69%	27%<br>21%	*Nature* **617**, 724-729 (2023)
Cu-Pd	70%	13.3%	*Nat. Catal.* **5**, 251 (2022)
Ag-Cu_2O	70%	-	*Nat. Synth.* **2**, 448 (2023)
Coordination Cu (I) polymer	61%	15%	*Adv. Mater.* **35**, 2209567 (2023)
2D Cu nanosheet	45%	-	*Nat. Catal.* **2,** 423–430 (2019)
Cu Nano-cubes	44%	15.5%	*PNAS* **118** e2010868118 (2021)
Cu NP	30%	10%	*Joule* **3,** 240–256 (2019)
OD-Cu	24%	5.8%	*Nat. Catal.***1,** 748–755 (2018)
$Ag_2Cu_2O_3$	25%	-	*Energy Environ. Sci.* **13,** 2993–3006 (2020)

Supplementary Table 2. EXAFS fitting parameters at the Cd K-edge for Cd foil and Cd-Cu sample.

Sample	Shell	CN [a]	R (Å) [b]	σ2 (Å2) [c]	ΔE0 (eV) [d]	R factor (%)
Cd foil	Cd-Cd	12*	2.951±0.012	0.0031	4.60±0.85	0.4
Cd-Cu	Cu-Cd	4.5±0.3	2.561±0.060	0.0057	5.98±2.88	1.1

[a] C*N*: coordination numbers; [b] *R*: bond distance; [c] σ^2: Debye-Waller factors; [d] ΔE_0: the inner potential correction. *R* factor: goodness of fit.

S_0^2 was set as 0.78 for Cd data, which was obtained from the experimental EXAFS fit of Cd foil reference by fixing CN as the known crystallographic value and was fixed to all the samples.

Supplementary Note 1

All the DFT calculations were carried out in the Vienna ab initio simulation package[1–3] with a plane wave pseudo-potential implementation[4]. The electronic exchange-correlation energy was treated by the spin-polarized generalized-gradient approximation (GGA) of Perdew-Burke-Ernzerhof (PBE)[4]. The electron-ion interaction was described by projector augmented wave (PAW) potentials[5,6]. The kinetic cut-off energy of 450 eV was used for the plane-wave expansion, with Brillouin zone meshed by gamma-point-centered Monkhorst-Pack[7] grids. The zero-damping DFT-D3 method of Grimme et al.[8] was used to describe the long-range Van de Waals interactions.

The (111) surface of face-centred cubic Cu was considered here using a 3x3x4 periodic cell, since the (111) surface is found to be the dominant surface in the Cu-based electrocatalyst material utilized in this work, which is proved by the XRD measurements. 15 Å of vacuum space was set among the periodic surfaces in the z-direction to decouple the interaction between them. Bimetallic Cd-Cu(111) surface was constructed by replacing one of the Cu atoms on the Cu(111) surface by Cd atom. To reduce the computational cost, all atoms in the two bottom-most layers were fixed during the structural optimization calculations while other metal atoms and the adsorbates were allowed to relax.

The computational hydrogen electrode model[9] was used to calculate the Gibbs free energy by correcting the electronic energies directly determined from DFT calculations with Zero-point energies, entropies and heat capacities, which are calculated from harmonic oscillator approximation at 298.15 K. Contributions to Gibbs free energies for each gas species and adsorbates are summarized in Supplementary Table 3. We note that the Gibbs free energies determined from our calculations for different for Cu(111) and Cd-Cu(111) surfaces provide a reasonable prediction of semiquantitative thermodynamic trends under electrochemical conditions, since we ignore the presence of transition state and charged intermediates on the surface.

A hexagonal charged water overlayer-that is, five water molecules and one hydronium (H_3O^+)[10] – was introduced to take into account of both field and solvation effects. To

optimize the structure of the charged water overlayer, Ab initio molecular dynamics simulations were conducted in canonical ensemble (NVT) with the Nose-Hoover thermostat[11–13] and a 1.0 fs time step at 300 K, as performed in our previous study[14]. Reaction intermediates were included in the optimized geometry from AIMD simulations, and again to perform DFT calculations. Charge-density difference[15,16] was applied to investigate the charge transfer between doped Cd atom and Cu atom.

Supplementary Table 3. Contributions to Gibbs free energies of gas species and adsorbates, including zero-point energy (ZPE), enthalpic temperature correction ($\int C_P \mathrm{d}T$), entropy (S). H_2 is referring to the energy used in computational hydrogen electrode model as described in Methods.

Species	$ZPE\ (eV)$	$\int C_P \mathrm{d}T\ (eV)$	$-TS$ (eV)	$G-E\ (eV)$
CO (g)	0.13	0.09	-0.68	-0.46
H_2O (l)	0.57	0.10	-0.67	0
H_2 (g)	0.27	0.08	-0.40	-0.04
C_2H_4 (g)	1.35	0.11	-0.73	0.73
CH_3COOH (l)	1.63	0.16	-0.95	0.84
OCCOH*	0.74	0.1	-0.17	0.67
CCOH*	0.59	0.09	-0.16	0.52
CHCOH*	0.91	0.09	-0.15	0.85
CCO*	0.35	0.08	-0.14	0.27
CHCO*	0.60	0.08	-0.12	0.56
H*	0.17	0.01	-0.01	0.17

FEM simulation

The simulation of cation concentration and electric field were performed in the COMSOL Multiphysics package based on the finite-element-method solver (https://www.comsol.com/). The electric double layer at the electrode-electrolyte interface was modelled using the Gouy-Chapman-Stern (GCS) model[17], which consists

of the stern layer and the diffuse layer. The Stern layer consists of a monolayer of surface-adsorbed hydrated cations on the electrode surface, with a thickness of d_s. The diffuse layer contains free anions and cations distributed according to the Poisson and Boltzmann law, which forms the concentration gradient away from the electrode surface.

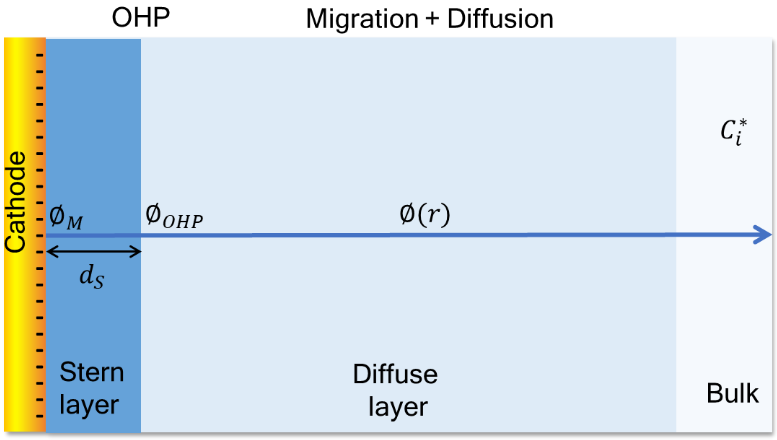

Supplementary Fig. 28 | Simulation domain.

Modified Poisson-Boltzmann (MPB) was applied to calculate the distribution of the cation and E-field at the electrode-electrolyte interface. The charge density at OHP, σ_{OHP}, is calculated according to:[18]

$$\sigma_{OHP} = \frac{\varepsilon_0 \varepsilon_r (\phi_M - \phi_{OHP})}{d_s} \tag{S1}$$

where ϕ_M and ϕ_{OHP} are the potentials at the electrode surface and OHP, respectively; d_s denotes the Stern layer thickness depending on the hydrated cation. The size of different hydrated cations is provided in Supplementary Table S5. Since there is no charge presented in the Stern layer, the potential is assumed to drop linearly from ϕ_M to ϕ_{OHP} in the Stern layer.

The distribution of ionic species in the diffuse layer obeys the well-known Poisson-Boltzmann equation:[19]

$$\nabla \cdot \left(\varepsilon_0 \varepsilon_r \nabla \phi(\boldsymbol{r})\right) = -\sum_i z_i F C_i^* e^{-\left(\frac{z_i F}{RT}\phi(r)\right)} \tag{S2}$$

where z_i and C_i^* are the charge and the bulk concentration of the ith ionic species,

respectively; T is the temperature of the electrolyte; F and R is the Faradaic constant and the ideal gas constant, respectively. The unevenly distributed charged ionic species could generate a non-uniform potential in the diffuse layer, which is denoted as $\phi(\boldsymbol{r})$.

To take into account the steric effect, the effective solvent diameter of the charged species is considered in PB equation, which results in that the distribution of the ionic species in the diffuse layer follows equation (S3) when symmetric electrolyte (such as KOH) is used:[20]

$$\nabla \cdot \left(\varepsilon_0 \varepsilon_r \nabla \phi(\boldsymbol{r})\right) = \frac{2eN_A C^* \sinh\left(\frac{F\phi(r)}{RT}\right)}{1 + 2N_A a^3 C^* \sinh^2\left(\frac{F\phi(r)}{2RT}\right)} \tag{S3}$$

Furthermore, to account for the dependence of the dielectric permittivity on E-field, Booth model, equation (S4), is employed.[21]

$$\varepsilon_\mathrm{r}(E;\beta) = n^2 + (\varepsilon_r(0) - n^2)\frac{3}{\beta E}\left[\coth(\beta E) - \frac{1}{\beta E}\right] \tag{S4}$$

In equation (S4), $E = |-\nabla\phi(r)|$ is the local E-field strength, n is the electrolyte refractive index, and β is a parameter given by:[22]

$$\beta = \frac{5\mu}{2kT}(n^2 + 2) \tag{S5}$$

where μ is the dipole moment of the water molecule.

The detailed simulation processes have been provided in our previous report.[23]

Supplementary Table 4. Diffusion-coefficients in m^2 s^{-1}.

Constant	Value	Reference
D_{H^+}	9.311×10^{-9}	10
D_{Li^+}	1.029×10^{-9}	10
D_{Na^+}	1.334×10^{-9}	10
D_{K^+}	1.958×10^{-9}	10
D_{Cs^+}	2.06×10^{-9}	10
D_{OH^-}	5.273×10^{-9}	10

Supplementary Table 5. Size of solvated cation in m.[24]

Constant	Value
α_{H^+}	0.56×10^{-9}
α_{Li^+}	0.764×10^{-9}
α_{Na^+}	0.716×10^{-9}
α_{K^+}	0.662×10^{-9}
α_{Cs^+}	0.658×10^{-9}
α_{OH^-}	0.6×10^{-9}

Supplementary Note 2

Techno-economic assessment of the COR system to produce acetic acid. Techno-economic assessment is performed using models adopted from that reported previously.[25,26] This model is used to calculate the plant-gate levelized cost (in US dollar) to produce one tonne of acetic acid from CO, based on the performance achieved using the catalyst and system developed (acetate FE = 86%, full-cell voltage = 2.04 V at a current density of 150 mA/cm^2, single-pass CO conversion = 90%) at a production capacity of 650 tonne acetic acid per day. We consider the costs for the electricity input, electrolyzer, catalyst and membrane replacement, liquid separation and electrolyte recovery, input chemicals, installation, balance of plant, as well as other operational costs. We estimate that the gas separation cost is low due to the low gas product FE and the high single-pass CO conversion and hence we do not include it in the assessment. The detailed description and assumptions of the model are listed below.

Electricity cost. We first calculate the total current require of acetic acid on a molar basis for a constant production capacity of 650 tonne.

$$Total\ current\ required\ [A] = \frac{production\ rate\left[\frac{mol}{s}\right] \times No.e^{-}\ transferred \times Faraday's\ constant}{FE_{acetate}}$$

$$Total\ current\ required\ [A] = \frac{\frac{650 \times 10^6 g}{60\frac{g}{mol} \times 86400\frac{s}{day}} \times 4 \times 96485\frac{sA}{mol}}{86\ \%} = 56\ 269\ 065\ A \qquad \text{(S6)}$$

Then, the power consumption can be calculated by multiplying the current with the cell potential of 2.04 V:

$$Power\ Consumed\ [W] = Total\ current\ needed\ [A] \times Cell\ voltage\ [V] = 56\ 269\ 065\ \ A\ \times 2.04\ V = 114\ 789\ kW \qquad (S7)$$

We can then find the electricity cost required to produce one tonne of acetic acid, assuming an electricity price of 2 cents per kWh:

$$Electricity\ cost\ \left[\frac{\$}{tonne_{acetic\ acid}}\right] = \frac{power\ Consumed\ [kW] \times 24[h] \times electricity\ price\ \left[\frac{\$}{kWh}\right]}{daily\ production\ [tonne]} = \frac{114\ 789\ \ kW \times 24\ h \times 0.02\frac{\$}{kWh}}{650\ tonne} \qquad \text{(S8)}$$

$$= \mathbf{84.77}\ \frac{\$}{\boldsymbol{tonne}_{acetic\ acid}}$$

Electrolyzer cost. We calculate the electrolyzer cost based on the DOE H2A analysis for central grid electrolysis where the electrolyzer cost for the stack component is \$250.25/kW with a reference current density of 175 mA/cm^2.[27] The one-time electrolyzer cost can be calculated as:

$$Electrolyzer\ cost\ [\$] = power\ consumed\ [kW] \times reference\ electrolyzer\ unit\ cost\ [\$/kW] \times \frac{reference\ current\ density\ [\frac{mA}{cm^2}]}{input\ current\ density\ [\frac{mA}{cm^2}]}$$

$$Electrolyzer\ cost\ [\$] = 114\,789\ kW \times 250.25 \frac{\$}{kW} \times \frac{175 \frac{mA}{cm^2}}{150 \frac{mA}{cm^2}} = \$\ 33\,513\,574 \tag{S9}$$

The one-time electrolyzer cost can be converted to a cost to generate one tonne of acetic acid. We assume the lifetime of the electrolyzer to be 20 years with no salvage value at the end of the plant's lifetime, as well as a plant capacity factor of 0.9 and a discount rate of 7%. The capital recovery factor (CRF) can be calculated.

$$CRF_{electrolyzer} = \frac{i(1+i)^{lifetime}}{(1+i)^{lifetime} - 1} = \frac{0.07(1.07)^{20}}{1.07^{20} - 1} = 0.094 \tag{S10}$$

The electrolyzer cost per tonne of acetic acid can be calculated using the CRF.

$$Electrolyzer\ cost\ \left[\frac{\$}{tonne_{acetic\ acid}}\right] = \frac{CRF_{electrolyzer} \times total\ electrolyzer\ cost\ [\$]}{capacity\ factor\ \times\ 365\ \left[\frac{day}{year}\right] \times production\ capacity \left[\frac{tonne\ acetic\ acid}{day}\right]} \tag{S11}$$

$$Electrolyzer\ cost\ \left[\frac{\$}{tonne_{acetic\ acid}}\right] = \frac{0.094 \times \$\ 33\,513\,574}{0.9 \times 365 \frac{day}{year} \times 650 \frac{tonne\ acetic\ acid}{day}} = \mathbf{14.82} \frac{\$}{\boldsymbol{tonne_{acetic\ acid}}}$$

Catalyst and membrane replacement cost. We assume the one-time catalyst and membrane replacement cost to be 5% of the total electrolyzer cost with a lifetime of 5 years and a discount rate of 7%. The total cost catalyst and membrane replacement cost can be calculated.

$$CRF_{catalyst\ and\ membrane} = \frac{i(1+i)^{lifetime}}{(1+i)^{lifetime} - 1} = \frac{0.07(1.07)^{5}}{1.07^{5} - 1} = 0.243 \tag{S12}$$

Similarly, the one-time catalyst and membrane replacement cost can be converted to a cost to generate one tonne of acetic acid.

$$Catalyst\ and\ membrane\ cost\ \left[\frac{\$}{tonne_{acetic\ acid}}\right] = \frac{CRF_{catalyst\ and\ membrane} \times total\ catalyst\ and\ membrane\ cost\ [\$]}{capacity\ factor\ \times\ 365\ \left[\frac{day}{year}\right] \times production\ capacity \left[\frac{tonne\ acetic\ acid}{day}\right]}$$

$$\textit{Catalyst and membrane cost}\ \left[\frac{\$}{tonne_{acetic\ acid}}\right] = \frac{0.243 \times 5\% \times \$\ 33\ 513\ 574}{0.9 \times 365\ \frac{day}{year} \times 650\frac{tonne_{acetic\ acid}}{day}}$$

$$= \mathbf{1.91}\ \frac{\$}{\boldsymbol{tonne_{acetic\ acid}}} \quad \text{(S13)}$$

Electrolyte recovery and liquid production separation. The acetate is assumed to accumulate in the electrolyzer until a final concentration of 45.6 wt% (7.6 M) is achieved.[28] The cost to protonate acetate to produce acetic acid and to recover the electrolyte is calculated using a similar method as reported.[28] To briefly describe the process, alkali acetate is first protonated with an HCl solution to produce acetic acid and alkali chloride salt. It is assumed that all alkali cations are bound to acetate. The alkali chloride salt is then subsequently converted back to alkali hydroxide and HCl via electrolysis. A typical electricity demand of NaCl electrolysis (2000 kWh/tonne NaOH) is assumed for this process.[29] An electricity price of 2 cent/kWh is used for the calculation. The electricity cost is assumed to be 50% of the total cost as reported for a typical chloralkali process.

Electricity usage (kWh/mol)	0.08 kWh/mol$_{\text{acetic acid}}$
Electricity usage (kWh/tonne)	1333 kWh/tonne$_{\text{acetic acid}}$
Cost (\$/tonne)	\$53.33/tonne$_{\text{acetic acid}}$

The separation cost is then studied using an ASPEN Plus model as described in **Supplementary Note 3**. The model estimates a capital cost of \$ 11 845 500 and an operating cost of \$ 56 433 per day for a production capacity of 650 tonne/day.

The capital cost can be converted to a cost to generate one tonne of acetic acid. We assume the lifetime of the electrolyzer to be 20 years with no salvage value at the end of the plant's lifetime, as well as a plant capacity factor of 0.9 and a discount rate of 7%.

$$\textit{Distillation unit cost}\ \left[\frac{\$}{tonne_{acetic\ acid}}\right]$$

$$= \frac{CRF_{distillation} \times \textit{distillation capital cost}\ [\$]}{\textit{capacity factor} \times 365\ \left[\frac{day}{year}\right] \times \textit{production capacity}\left[\frac{\textit{tonne acetic acid}}{day}\right]}$$

$$\textit{Distillation unit cost}\ \left[\frac{\$}{tonne_{acetic\ acid}}\right] = \frac{0.094 \times \$\ 11\ 845\ 500}{0.9 \times 365\ \frac{day}{year} \times 650\frac{\textit{tonne acetic acid}}{day}}$$

$$= \mathbf{5.24}\ \frac{\$}{\boldsymbol{tonne_{acetic\ acid}}} \quad \text{(S14)}$$

The operating cost can also be converted to a cost to generate one tonne of acetic acid.

$$\textit{Distillation operating cost}\left[\frac{\$}{tonne_{acetic\ acid}}\right]=\frac{\textit{Distillation operating cost}\left[\frac{\$}{day}\right]}{\textit{production capacity}\left[\frac{tonne_{acetic\ acid}}{day}\right]}$$

$$=\frac{\$\ 56\ 433}{650\,\frac{tonne_{acetic\ acid}}{day}}=\mathbf{86.82}\ \frac{\$}{\boldsymbol{tonne_{acetic\ acid}}} \quad \text{(S15)}$$

Input chemicals costs. The costs of input CO and water are calculated based on a CO price of \$180 per tonne and a water price of \$5 per tonne.[30] We estimate that the energy to pressurize CO to 8 bar is ~1% of the electrolyzer electricity input and hence the cost of pressurization is not included.

$$\textit{CO cost}\left[\frac{\$}{tonne_{acetic\ acid}}\right]$$

$$=1\ tonne_{acetic\ acid}\times\frac{\textit{molar mass}_{CO}\left[\frac{g}{mol}\right]}{\textit{molar mass}_{acetic\ acid}\left[\frac{g}{mol}\right]}\times \textit{molar ratio}\left[\frac{CO}{acetic\ acid}\right]$$

$$\times\ \textit{CO unit price}\left[\frac{\$}{tonne}\right]$$

$$\textit{CO cost}\left[\frac{\$}{tonne_{acetic\ acid}}\right]=1\ tonne_{acetic\ acid}\times\frac{28\frac{g}{mol}}{60\frac{g}{mol}}\times 2\times 180\,\frac{\$}{tonne}$$

$$=\mathbf{168}\ \frac{\$}{\boldsymbol{tonne_{acetic\ acid}}} \quad \text{(S16)}$$

$$H_2O\ \textit{cost}\left[\frac{\$}{tonne_{acetic\ acid}}\right]$$

$$=1\ tonne_{acetic\ acid}\times\frac{\textit{molar mass}_{H_2O}\left[\frac{g}{mol}\right]}{\textit{molar mass}_{acetic\ acid}\left[\frac{g}{mol}\right]}\times \textit{molar ratio}\left[\frac{H_2O}{acetic\ acid}\right]$$

$$\times\ H_2O\ \textit{unit price}\left[\frac{\$}{tonne}\right]$$

$$H_2O\ \textit{cost}\left[\frac{\$}{tonne_{acetic\ acid}}\right]=1\ tonne_{acetic\ acid}\times\frac{18\frac{g}{mol}}{60\frac{g}{mol}}\times 2\times 5\,\frac{\$}{tonne}$$

$$=\mathbf{3}\ \frac{\$}{\boldsymbol{tonne_{acetic\ acid}}} \quad \text{(S17)}$$

Installation costs. We assume a Lang factor of 1 for the equipment installation cost. The total capital costs are the sum of the capital cost of the electrolyzer, membrane and catalyst, and the distillation unit.

$$Installation\ cost\ \left[\frac{\$}{tonne_{acetic\ acid}}\right] = Lang\ factor \times total\ capital\ cost\ \left[\frac{\$}{tonne_{acetic\ acid}}\right]$$
$$= 1 \times (14.82 + 1.91 + 5.24) = \mathbf{21.97}\frac{\$}{\boldsymbol{tonne_{acetic\ acid}}} \tag{S18}$$

Balance of plant (BoP). We assume the balance of plant is 50% of the total capital costs. The total capital costs are the sum of the capital cost of the electrolyzer, membrane and catalyst, and the distillation unit.

$$BoP\ \left[\frac{\$}{tonne_{acetic\ acid}}\right] = 50\% \times total\ capital\ cost\ \left[\frac{\$}{tonne_{acetic\ acid}}\right]$$
$$= 50\% \times (14.82 + 1.91 + 5.24) = \mathbf{10.98}\frac{\$}{\boldsymbol{tonne_{acetic\ acid}}} \tag{S19}$$

Other operational costs. We assume other operational costs (such as labor and maintenance) to be 10% of the electrolyzer electricity cost.

$$Operational\ cost\ \left[\frac{\$}{tonne_{acetic\ acid}}\right] = 10\% \times Electricity\ cost\ \left[\frac{\$}{tonne_{acetic\ acid}}\right]$$
$$= 10\% \times\ 84.76\ \frac{\$}{tonne_{acetic\ acid}} = \mathbf{8.48}\frac{\$}{\boldsymbol{tonne_{acetic\ acid}}} \tag{S20}$$

Plant-gate levelized cost. The plant-gate levelized cost to produce 1 tonne of acetic acid can be calculated by summing up all costs discussed earlier. The market price for acetic acid is assumed to be \$600/tonne.[30]

$$Plant - gate\ levelized\ cost\ \left[\frac{\$}{tonne_{acetic\ acid}}\right]$$
$$= (84.77 + 14.82 + 1.91 + 53.33 + 5.24 + 86.82 + 168 + 3 + 21.97 + 10.98$$
$$+ 8.48)\frac{\$}{tonne_{acetic\ acid}} = \mathbf{459.31}\frac{\$}{\boldsymbol{tonne_{acetic\ acid}}} \tag{S21}$$

Supplementary Note 3

Aspen Plus simulation to model the acetic acid separation cost. The distillation cost is studied using an ASPEN Plus model with the Economic Analyzer plugin using the NRTL-HOC method. Glacial acetic acid (>98 wt%) is produced at a scale of 27.384 tonne/hour by intaking 45.6 wt% acetic acid.[31] Azeotropic distillation is used to achieve efficient separation due to the close boiling points of acetic acid and water.[32] In the modeled azeotropic distillation process (Figure S23), n-propyl acetate is used as the entrainer to separate acetic acid from water.[33] The process involves feeding a mixture of water and acetic acid into a RadFrac column in ASPEN software to produce glacial acetic acid, which is collected at the bottom of this dehydration column. The distillate is then directed to a decanter to separate n-propyl acetate from water, where the top entrainer-rich layer is recycled and combined with a make-up n-propyl acetate stream to serve as the entrainer stream. The remaining n-propyl acetate in the bottom water layer of the decanter is extracted using another RadFrac column. The residual n-propyl acetate is separated into the distillate and recycled back to the first dehydration column, completing the water purification process.

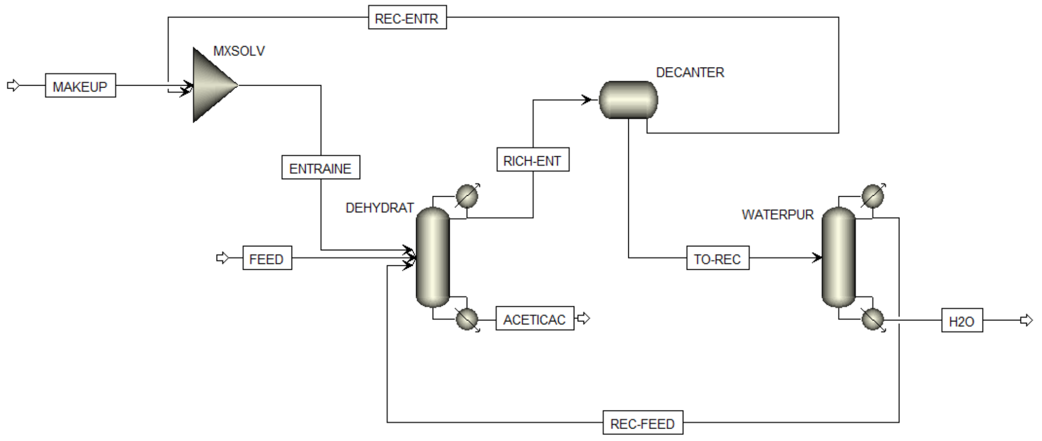

Supplementary Fig. 29 | Azeotropic distillation process for separating acetic acid from water using ASPEN PLUS.

References

1 Kresse, G.; Hafner, Ab initio molecular dynamics for liquid metals. *J. Phys. Rev. B* ***47***, 558-561, (1993).

2 Kresse, G.; Furthmüller, Efficiency of ab-initio total energy calculations for metals and semiconductors using a plane-wave basis set. *J. Comput. Mater. Sci.* ***6***, 15-50, (1996).

3 Kresse, G.; Furthmüller, Efficient iterative schemes for ab initio total-energy calculations using a plane-wave basis set. *J. Phys. Rev. B* ***54***, 11169-11186, (1996).

4 Payne, M. C.; Teter, M. P.; Allan, D. C.; Arias, T. A.; Joannopoulos, Iterative minimization techniques for ab initio total-energy calculations: molecular dynamics and conjugate gradients. *J. D. Rev. Mod. Phys.* ***64***, 1045-1097 (1992).

5 Blöchl, P. E. Projector augmented-wave method. *Phys. Rev. B* ***50***, 17953-17979, (1994).

6 Joubert, D. From ultrasoft pseudopotentials to the projector augmented-wave method. *Phys. Rev. B - Condens. Matter Mater. Phys.* ***59***, 1758-1775 (1999).

7 Monkhorst, H. J.; Pack, J. D. Special points for Brillouin-zone integrations. *Phys. Rev. B* ***13***, 5188-5192 (1976).

8 Grimme, S.; Antony, J.; Ehrlich, S.; Krieg, H. A consistent and accurate ab initio parametrization of density functional dispersion correction (DFT-D) for the 94 elements H-Pu. *J. Chem. Phys.* ***132***, 154104 (2010).

9 Nørskov, J. K.; Rossmeisl, J.; Logadottir, A.; Lindqvist, L.; Kitchin, J. R.; Bligaard, T.; Jónsson, H. Origin of the Overpotential for Oxygen Reduction at a Fuel-Cell Cathode. *J. Phys. Chem. B* ***108***, 17886-17892, (2004).

10 Montoya, J. H.; Shi, C.; Chan, K.; Nørskov, J. K. Theoretical Insights into a CO Dimerization Mechanism in CO_2 Electroreduction. *J. Phys. Chem. Lett.* 2015, *6*, 2032-2037 (2015).

11 Nosé, S. A unified formulation of the constant temperature molecular dynamics methods *J. Chem. Phys.* ***81***, 511-519 (1984).

12 Hoover, W. G. Canonical dynamics: Equilibrium phase-space distributions. *Phys. Rev. A* ***31***, 1695-1697 (1985).

13 Nosé, S. Constant Temperature Molecular Dynamics Methods. *Prog. Theor. Phys. Suppl.* ***103***, 1-46 (1991).

14 Xie, Y.; Ou, P.; Wang, X.; Xu, Z.; Li, Y. C.; Wang, Z.; Huang, J. E.; Wicks, J.; McCallum, C.; Wang, N.; Wang, Y.; Chen, T.; Lo, B. T. W.; Sinton, D.; Yu, J. C.; Wang, Y.; Sargent, E. H. High carbon utilization in CO_2 reduction to multi-carbon products in acidic media. *Nat. Catal.* **5**, 564-570 (2022).

15 Bader, R. F. W. A quantum theory of molecular structure and its applications. *Chem. Rev.* ***91***, 893-928 (1991).

16 Tang, W.; Sanville, E.; Henkelman, G. A grid-based Bader analysis algorithm without lattice bias. *J. Phys. Condens. Matter* ***21***, 084204 (2009).

17 R. Grassi, A. Daghe'Iti, S. Trasatti. Application of the gouy-chapman-stern-gbahame model of the eleci'rical double layer to the determination of single ion acltvities of kf aqueous solutions. *J. Electroanal. Chem*, **226**, 341-349, (1987).

18 Henri Orland. Steric Effects in Electrolytes: A modified poisson-boltzmann equation. *Pyhs. Rev. Lett.*, **79**, 435-438, (1997).

19 Debye, P. and Hückel E. (1923) Zur Theorie der Elektrolyte. Physikalische Zeitschrift, 9,

185-206.

20 Xing, X. Poisson-Boltzmann theory for two parallel uniformly charged plates. *Phys. Rev. E. Stat. Nonlin. Soft. Matter. Phys.* **83**, 041410 (2011).

21 Elena Castellani. Reductionism, emergence, and effective field theories. *Stud. Hist. Philos. M. P.* **33**, 251–267, (2022).

22 Brivio, I.; Trott, M. The standard model as an effective field theory. *Phy. Rep.* **793**, 1-98 (2019).

23 Li, F., Zhou, C. & Klinkova, A. Simulating electric field and current density in nanostructured electrocatalysts. *Phys. Chem. Chem. Phys.* **24**, 25695-25719, (2022).

24 Nightingale, E. R. Phenomenological theory of ion solvation. effective radii of hydroated ions. *Phys. Chem*. **63**, 1381-1387 (1959).

25 Wang, X. *et al.* Efficient electrosynthesis of n-propanol from carbon monoxide using a Ag–Ru–Cu catalyst. *Nat. Energy* **7**, 170-176, (2022).

26 Sisler, J. *et al.* Ethylene Electrosynthesis: A Comparative Techno-economic Analysis of Alkaline vs Membrane Electrode Assembly vs CO_2-CO-C_2H_4 Tandems. *ACS Energy Lett.* **6**, 997-1002, (2021).

27 Jouny, M., Luc, W. & Jiao, F. General Techno-Economic Analysis of CO_2 Electrolysis Systems. *Ind. Eng. Chem. Res.* **57**, 2165-2177, (2018).

28 Overa, S. *et al.* Enhancing acetate selectivity by coupling anodic oxidation to carbon monoxide electroreduction. *Nat. Catal.* **5**, 738-745, (2022).

29 Kumar, A., Du, F. & Lienhard, J. H. Caustic Soda Production, Energy Efficiency, and Electrolyzers. *ACS Energy Lett.* **6**, 3563-3566, (2021).

30 Huang, Z., Grim, R. G., Schaidle, J. A. & Tao, L. The economic outlook for converting CO_2 and electrons to molecules. *Energy & Environ. Sci.* **14**, 3664-3678, (2021).

31 Dimian, A. C., Bildea, C. S. & Kiss, A. A. Applications in Design and Simulation of Sustainable Chemical Processes 483-519 (2019).

32 Lloyd Berg. *et al.* Dethydration of acetic acid by azeotropic distillation. U.S. Patent 5, 160, 412[P]. 1992-3-10.

33 Federico Galli. *et al.* Simulation of the Water-Acetic Acid Separation via Distillation Using Different Entrainers: an Economic Comparison. *Chem. Eng. trans.* 57, 1159-1164, (2017) ISBN 978-88-95608- 48-8; ISSN 2283-9216.
