# Supplementary material for: Site-selective protonation enables efficient carbon monoxide electroreduction to acetate
Source: Nat Commun. 2024 Jan 19;15:616. doi: 10.1038/s41467-024-44727-z (PMC10798983; doi:10.1038/s41467-024-44727-z)
Supplement: Supplementary file 3 — Description of Additional Supplementary Files [file 41467_2024_44727_MOESM3_ESM.pdf]

Description of Additional Supplementary Files

Supplementary Data 1 The atomic coordination of the optimized models
