# Supplementary material for: Site-selective protonation enables efficient carbon monoxide electroreduction to acetate
Source: Nat Commun. 2024 Jan 19;15:616. doi: 10.1038/s41467-024-44727-z (PMC10798983; doi:10.1038/s41467-024-44727-z)
Supplement: Supplementary file 4 — Supplementary Data 1 [file 41467_2024_44727_MOESM4_ESM.pdf]

Cu111 surface
55

| | | | |
|---|---|---|---|
| Cu | 0.299505568669981 | 0.469397725665565 | 0.000000000000000 |
| Cu | -0.984205260760016 | 2.692850104464759 | 0.000000000000000 |
| Cu | -2.267954601900021 | 4.916369187502353 | 0.000000000000000 |
| Cu | -2.266042764194945 | 4.901756877390661 | 6.196539094987749 |
| Cu | 0.307864650816695 | 0.456512281919643 | 6.191104726671907 |
| Cu | -0.977668486921142 | 2.677630468865982 | 6.173111834694740 |
| Cu | 0.299505568670020 | 1.951699311531695 | 2.096321626389947 |
| Cu | -0.984243772469984 | 4.175218394569288 | 2.096321626389947 |
| Cu | -2.267954601899981 | 6.398670773368481 | 2.096321626389947 |
| Cu | 0.304933726998710 | 3.424222380602397 | 4.135485683251066 |
| Cu | 1.585352656586205 | 1.201284638143148 | 4.139506693011779 |
| Cu | -0.983181942967122 | 5.648384157813748 | 4.137850252673435 |
| Cu | 2.867004250950020 | 0.469397725665565 | 0.000000000000000 |
| Cu | 1.583293421520022 | 2.692850104464759 | 0.000000000000000 |
| Cu | 0.299544080380018 | 4.916369187502353 | 0.000000000000000 |
| Cu | 1.587350621897294 | 2.678080202762694 | 6.192077247453679 |
| Cu | 2.875124920218928 | 0.449247245065715 | 6.183992379458881 |
| Cu | 0.307341773296147 | 4.902935060006529 | 6.181419028853529 |
| Cu | 2.867004250950004 | 1.951699311531695 | 2.096321626389947 |
| Cu | 1.583254909810000 | 4.175218394569288 | 2.096321626389947 |
| Cu | 0.299544080380002 | 6.398670773368481 | 2.096321626389947 |
| Cu | 4.153974764404047 | 1.200707093283012 | 4.133585962582299 |
| Cu | 1.585392414079675 | 5.645814648793432 | 4.130699223749454 |
| Cu | 2.870626497468243 | 3.422726196233243 | 4.136168056572353 |
| Cu | 5.434425909810021 | 0.469397725665565 | 0.000000000000000 |
| Cu | 4.150715080379969 | 2.692850104464759 | 0.000000000000000 |
| Cu | 2.866965739240020 | 4.916369187502353 | 0.000000000000000 |
| Cu | 5.444195408253024 | 0.445684294659922 | 6.169171150047739 |
| Cu | 4.157499227675984 | 2.674193160212503 | 6.187222609941348 |
| Cu | 5.434425909810005 | 1.951699311531695 | 2.096321626389947 |
| Cu | 4.150676568670002 | 4.175218394569288 | 2.096321626389947 |
| Cu | 2.866965739240004 | 6.398670773368481 | 2.096321626389947 |
| Cu | 5.434404301517859 | 3.426975193975258 | 4.135882773309732 |
| Cu | 4.155823011345757 | 5.649202247073569 | 4.138603998135595 |
| Cu | 6.724130114121771 | 1.201983256277850 | 4.131717119878917 |
| Cu | 2.872566030977437 | 4.894034066667883 | 6.176980808945215 |
| H | -0.768904545684727 | 4.182217311135750 | 8.319521211733292 |
| H | -1.525023239466118 | 5.288169425551418 | 9.779551639581213 |
| H | 0.181544014537962 | 4.059527024163036 | 9.635018339554234 |
| H | 2.256749623385843 | 0.336049443765552 | 9.789417633230823 |
| H | 2.061411079584340 | 3.156791390335509 | 9.860782117684973 |
| H | 2.741824577939626 | 1.699556899167983 | 8.309086714393185 |
| H | 2.155818494761347 | 4.736337292219015 | 9.840868471271012 |
| H | 3.876896956064605 | 1.698373024433935 | 9.404025498266366 |
| H | 5.562289556913028 | 1.686462778142393 | 8.240218236528472 |
| H | 3.261221501531955 | 5.981011887287618 | 8.303262577394509 |
| H | 6.556812473463399 | 0.296845760937860 | 9.397288307791776 |

| | | | |
|---|---|---|---|
| H | 6.053214775419382 | 2.574052703564221 | 9.424416703881109 |
| H | -3.034080368880463 | 6.132866742086640 | 9.798418072877027 |
| O | -0.815933569736821 | 4.112428170583688 | 9.312848715690919 |
| O | -2.053466168569515 | 6.143270725789946 | 10.129997871040743 |
| O | 1.586105077613060 | 3.981689681232174 | 10.135168409966298 |
| O | 2.876640420839241 | 1.655244216835622 | 9.298235052768849 |
| O | 5.583344924991860 | 1.706764191175494 | 9.241885107363048 |
| O | 3.212204489007821 | 6.103836971333453 | 9.293993476198507 |

Cu111_H
56

| | | | |
|---|---|---|---|
| Cu | 0.299505472692490 | 0.469397638141238 | 0.000000000000000 |
| Cu | -0.984205005895600 | 2.692849409262957 | 0.000000000000000 |
| Cu | -2.267953992333149 | 4.916367877936429 | 0.000000000000000 |
| Cu | -2.254012818323810 | 4.910631474919341 | 6.236169469094603 |
| Cu | 0.306668683670200 | 0.473266463016237 | 6.192740051906956 |
| Cu | -0.967452446930910 | 2.673438560654516 | 6.172941103128990 |
| Cu | 0.299505480394830 | 1.951698783313465 | 2.096322006690440 |
| Cu | -0.984243621577822 | 4.175217452099605 | 2.096322006690440 |
| Cu | -2.267954046249530 | 6.398669129835413 | 2.096322006690440 |
| Cu | 0.305243708384020 | 3.426329169729693 | 4.137292810841540 |
| Cu | 1.585834750774589 | 1.202943989432973 | 4.141763211575552 |
| Cu | -0.997761109112680 | 5.642243363745411 | 4.148150000149164 |
| Cu | 2.867003507186309 | 0.469397638141238 | 0.000000000000000 |
| Cu | 1.583293028598220 | 2.692849409262957 | 0.000000000000000 |
| Cu | 0.299544042160671 | 4.916367877936429 | 0.000000000000000 |
| Cu | 1.576476380563309 | 2.673838732624200 | 6.192740051906956 |
| Cu | 2.872549265249261 | 0.451387491483026 | 6.185714680276215 |
| Cu | 0.306630171969570 | 4.903160401922851 | 6.182733994437712 |
| Cu | 2.867003568805031 | 1.951698783313465 | 2.096322006690440 |
| Cu | 1.583254466832379 | 4.175217452099605 | 2.096322006690440 |
| Cu | 0.299544042160671 | 6.398669129835413 | 2.096322006690440 |
| Cu | 4.153641009813231 | 1.218686092480834 | 4.143040697023917 |
| Cu | 1.600315127104449 | 5.639308371299200 | 4.141550343460195 |
| Cu | 2.867735475973164 | 3.423927737686255 | 4.133673520656968 |
| Cu | 5.434424579897590 | 0.469397638141238 | 0.000000000000000 |
| Cu | 4.150714101309501 | 2.692849409262957 | 0.000000000000000 |
| Cu | 2.866965114871952 | 4.916367877936429 | 0.000000000000000 |
| Cu | 5.445284771642488 | 0.449252936362095 | 6.173366818070764 |
| Cu | 4.157645980203867 | 2.660698047548287 | 6.226163411625359 |
| Cu | 5.434424410446106 | 1.951698783313465 | 2.096322006690440 |
| Cu | 4.150675308473455 | 4.175217452099605 | 2.096322006690440 |
| Cu | 2.866964883801747 | 6.398669129835413 | 2.096322006690440 |
| Cu | 5.437043437159151 | 3.427062716060973 | 4.132183486427352 |
| Cu | 4.155643410282808 | 5.655050474347061 | 4.135163874220689 |
| Cu | 6.723411099853268 | 1.203010686984728 | 4.133460652541611 |
| Cu | 2.860495314766424 | 4.904894678347341 | 6.217221950200184 |
| H | 4.145168231562617 | 4.158474618958533 | 7.103268012796284 |
| H | -0.761568891511760 | 4.198830613341819 | 8.328446172613532 |

| | | | |
|---|---|---|---|
| H | -1.529607687110640 | 5.290578637724044 | 9.794828836479017 |
| H | 0.176537504748138 | 4.063354438156665 | 9.649637773102834 |
| H | 2.256054042438792 | 0.338257110659567 | 9.811647099418479 |
| H | 2.054714640474948 | 3.157044104733544 | 9.866998025201594 |
| H | 2.731018865564432 | 1.673875717957527 | 8.318440732523561 |
| H | 2.149915722330281 | 4.736733581044747 | 9.874449111774108 |
| H | 3.871773680343758 | 1.701424601739161 | 9.407795643999785 |
| H | 5.575608362723238 | 1.677944635487790 | 8.257128435063441 |
| H | 3.235675986507939 | 5.998443776762146 | 8.354205875689230 |
| H | 6.546834960167058 | 0.315510970897210 | 9.449308971392998 |
| H | 6.048686162583220 | 2.589524602462895 | 9.431213542339277 |
| H | -3.037764445547281 | 6.139923532055897 | 9.832296881989659 |
| O | -0.817950032787591 | 4.120786840155285 | 9.320723643463968 |
| O | -2.054676298225801 | 6.145326754153634 | 10.153759925056010 |
| O | 1.579210977038773 | 3.977439219891289 | 10.153973091216534 |
| O | 2.871547972586391 | 1.650862821340262 | 9.307311781304781 |
| O | 5.581346825633802 | 1.718701001953426 | 9.258134158600651 |
| O | 3.198974304998189 | 6.120712869488631 | 9.343077563138664 |

Cu111_2H
57

| | | | |
|---|---|---|---|
| Cu | 0.299505472692490 | 0.469397638141238 | 0.000000000000000 |
| Cu | -0.984205005895600 | 2.692849409262957 | 0.000000000000000 |
| Cu | -2.267953992333149 | 4.916367877936429 | 0.000000000000000 |
| Cu | -2.253743328847482 | 4.913633271644062 | 6.235956281645140 |
| Cu | 0.302740463247750 | 0.469931245236965 | 6.196145835307959 |
| Cu | -0.976310114968790 | 2.663299465520235 | 6.169322132278524 |
| Cu | 0.299505480394830 | 1.951698783313465 | 2.096322006690440 |
| Cu | -0.984243621577822 | 4.175217452099605 | 2.096322006690440 |
| Cu | -2.267954046249530 | 6.398669129835413 | 2.096322006690440 |
| Cu | 0.301623516096718 | 3.425795602656499 | 4.129415668704207 |
| Cu | 1.585603649761449 | 1.216151372060642 | 4.151982137160153 |
| Cu | -0.981932884879491 | 5.634972670232339 | 4.164968263088627 |
| Cu | 2.867003507186309 | 0.469397638141238 | 0.000000000000000 |
| Cu | 1.583293028598220 | 2.692849409262957 | 0.000000000000000 |
| Cu | 0.299544042160671 | 4.916367877936429 | 0.000000000000000 |
| Cu | 1.572509798635861 | 2.662165547106603 | 6.231485880911130 |
| Cu | 2.875245038079320 | 0.460992872794825 | 6.188694748735444 |
| Cu | 0.298966266520799 | 4.914833987665781 | 6.217435137649646 |
| Cu | 2.867003568805031 | 1.951698783313465 | 2.096322006690440 |
| Cu | 1.583254466832379 | 4.175217452099605 | 2.096322006690440 |
| Cu | 0.299544042160671 | 6.398669129835413 | 2.096322006690440 |
| Cu | 4.155065861861970 | 1.215551014049782 | 4.146446778470086 |
| Cu | 1.603627503070954 | 5.640909059177938 | 4.139421726173451 |
| Cu | 2.872356729853131 | 3.423260582067306 | 4.137931393898669 |
| Cu | 5.434424579897590 | 0.469397638141238 | 0.000000000000000 |
| Cu | 4.150714101309501 | 2.692849409262957 | 0.000000000000000 |
| Cu | 2.866965114871952 | 4.916367877936429 | 0.000000000000000 |
| Cu | 5.451177196629831 | 0.459725505914306 | 6.174857171634484 |

| | | | |
|---|---|---|---|
| Cu | 4.176016334837452 | 2.658496801546024 | 6.229569812405635 |
| Cu | 5.434424410446106 | 1.951698783313465 | 2.096322006690440 |
| Cu | 4.150675308473455 | 4.175217452099605 | 2.096322006690440 |
| Cu | 2.866964883801747 | 6.398669129835413 | 2.096322006690440 |
| Cu | 5.439200261845914 | 3.428796999155887 | 4.131970320266830 |
| Cu | 4.139853975034494 | 5.643710863303727 | 4.147511417092034 |
| Cu | 6.725298384998491 | 1.200008990316341 | 4.132822069484482 |
| Cu | 2.876438777561408 | 4.897423998905762 | 6.290243207474518 |
| H | 4.215721759544858 | 4.151670694910569 | 7.142652105523480 |
| H | -0.768963191949101 | 4.228580936567961 | 8.371875589801180 |
| H | -1.542124001368899 | 5.321062513951889 | 9.839535419780923 |
| H | 0.161903378155853 | 4.084299383818843 | 9.696473612359696 |
| H | 2.250546722904240 | 0.367807081083978 | 9.847625430033613 |
| H | 2.040850305010706 | 3.182124958976318 | 9.923626734478239 |
| H | 2.718348404373230 | 1.693953722184915 | 8.349948002447530 |
| H | 2.133163109449210 | 4.759346405748661 | 9.942361065923196 |
| H | 3.861183043545080 | 1.726305269940760 | 9.436322845464524 |
| H | 5.561821162344188 | 1.690218379128914 | 8.283526401862163 |
| H | 3.244726359393431 | 6.024525167655886 | 8.407002447954886 |
| H | 6.539864507953341 | 0.343593441763305 | 9.491674409915850 |
| H | 6.034706484575590 | 2.610936711145090 | 9.452927942243464 |
| H | -3.046622171352711 | 6.171007772965022 | 9.882113385796085 |
| H | 1.526565382147141 | 4.157073917008305 | 7.124556676469759 |
| O | -0.829542073933071 | 4.141265016352357 | 9.361172992192387 |
| O | -2.060067797671878 | 6.176143811300830 | 10.196763606012944 |
| O | 1.564961578484612 | 3.998517754099221 | 10.220394713090839 |
| O | 2.860687557473629 | 1.676210359063300 | 9.338393974955189 |
| O | 5.568945861621268 | 1.738712208572726 | 9.283681334184038 |
| O | 3.194815133758230 | 6.149128894817722 | 9.394383781840597 |

Cu111_CCO
58

| | | | |
|---|---|---|---|
| C | 4.180567603752883 | 4.199231132528968 | 7.516479522786674 |
| C | 4.021503557695889 | 4.023644151281427 | 8.781589569739193 |
| Cu | 0.299505568669981 | 0.469397725665565 | 0.000000000000000 |
| Cu | -0.984205260760016 | 2.692850104464759 | 0.000000000000000 |
| Cu | -2.267954601900021 | 4.916369187502353 | 0.000000000000000 |
| Cu | -2.197607796434825 | 4.958894226951364 | 6.278002420296040 |
| Cu | 0.314859637878656 | 0.507018476682484 | 6.172477264202030 |
| Cu | -0.953288882267777 | 2.685624232970378 | 6.162293692214164 |
| Cu | 0.299505568670020 | 1.951699311531695 | 2.096321626389947 |
| Cu | -0.984243772469984 | 4.175218394569288 | 2.096321626389947 |
| Cu | -2.267954601899981 | 6.398670773368481 | 2.096321626389947 |
| Cu | 0.303908969890796 | 3.450236388419997 | 4.134043938704776 |
| Cu | 1.589116850953263 | 1.218381139998555 | 4.127654289609561 |
| Cu | -1.005492110337261 | 5.647300719621490 | 4.141431950747678 |
| Cu | 2.867004250950020 | 0.469397725665565 | 0.000000000000000 |
| Cu | 1.583293421520022 | 2.692850104464759 | 0.000000000000000 |
| Cu | 0.299544080380018 | 4.916369187502353 | 0.000000000000000 |

```
Cu       1.568953152594550       2.700467881022119       6.172105481847392
Cu       2.865723618581766       0.485936741173672       6.142131578400078
Cu       0.309621263173411       4.939333209700264       6.172108228393799
Cu       2.867004250950004       1.951699311531695       2.096321626389947
Cu       1.583254909810000       4.175218394569288       2.096321626389947
Cu       0.299544080380002       6.398670773368481       2.096321626389947
Cu       4.155206222092565       1.246568268201814       4.134386901379451
Cu       1.614467120826684       5.645441016864645       4.140359005119101
Cu       2.875918519763914       3.444273407214813       4.151678771624371
Cu       5.434425909810021       0.469397725665565       0.000000000000000
Cu       4.150715080379969       2.692850104464759       0.000000000000000
Cu       2.866965739240020       4.916369187502353       0.000000000000000
Cu       5.442539442410549       0.478882406016374       6.143602736269137
Cu       4.161694201686214       2.644830538713388       6.257297695638684
Cu       5.434425909810005       1.951699311531695       2.096321626389947
Cu       4.150676568670002       4.175218394569288       2.096321626389947
Cu       2.866965739240004       6.398670773368481       2.096321626389947
Cu       5.435276791579442       3.442858771030325       4.147796424826735
Cu       4.158131770738653       5.659055601925310       4.149407876027304
Cu       6.719734047713755       1.213082098369943       4.128405088069668
Cu       2.830826450475908       4.965672935767925       6.271234213955275
H       -0.736133972615517       3.968183415165859       8.434335335268154
H       -0.946734780830344       4.875396234971539      10.380892258185192
H        0.350986373133711       3.285372839320865       9.400277222585146
H       -0.547936542896904       6.441862140950594      10.203218275790608
H        1.805535417734310       1.843389932915237       8.718612517631867
H        3.854827910493781       0.253003104699507       8.290604358136969
H        2.415563012423559       2.665250468776276       9.886246482609847
H        4.947917019170127       0.740916113173883       9.324318990636977
H        6.814392583300114       0.763464351052882       8.422160384174534
H        3.859036798094019       5.412150394382017      10.986400165527813
H       -0.028698478350682       0.244406107551045      10.563602687868416
H       -1.064302118351558       1.997399377149502       9.375409792838095
H       -2.061739395253580       6.041142132629758      10.990476223546686
O       -0.596701047064816       3.660135859441190       9.363196210494158
O       -1.031429260602258       5.767699363047024      10.860638524629412
O        1.569652396105175       2.270861007105839       9.569995406151730
O        3.956222010801638       0.585750231763222       9.216507374902255
O        6.550790540903453       0.999239773305730       9.346497134377794
O       -3.451037568599435       6.288435674048444      11.204291664715683
O        3.844146286742528       3.835589333307945       9.969502840133490

Cu111_CCOH
59

C        2.820282360759073       2.070329241085408       7.781405553644429
C        2.813503221947626       3.410675081911068       7.600330960175086
Cu       2.879536133453703       4.875175120843865       6.299559899521337
Cu       0.258542462880322       0.436182602121381       6.172325703911442
Cu      -2.248082052667982       4.945258203086261       6.136252687614927
```

| | | | |
|---|---|---|---|
| Cu | 0.299505568670004 | 0.469397725665535 | 0.000000000000000 |
| Cu | -0.984205260760010 | 2.692850104464757 | 0.000000000000000 |
| Cu | -2.267954601900009 | 4.916369187502360 | 0.000000000000000 |
| Cu | -0.999938426912688 | 2.701819970476265 | 6.143735669499209 |
| Cu | 0.299505568670032 | 1.951699311531667 | 2.096321626389977 |
| Cu | -0.984243772469967 | 4.175218394569270 | 2.096321626389977 |
| Cu | -2.267954601899981 | 6.398670773368493 | 2.096321626389977 |
| Cu | 1.575957543340048 | 1.209231503739722 | 4.139346898276465 |
| Cu | -0.988481944258396 | 5.698670445113711 | 4.129589869923771 |
| Cu | 0.310456937029311 | 3.430173882120827 | 4.127607367728534 |
| Cu | 2.865136849870968 | 0.530314366425275 | 6.268727296931321 |
| Cu | 2.867004250950002 | 0.469397725665535 | 0.000000000000000 |
| Cu | 1.583293421519988 | 2.692850104464757 | 0.000000000000000 |
| Cu | 0.299544080379989 | 4.916369187502360 | 0.000000000000000 |
| Cu | 1.441498385838331 | 2.665318883291907 | 6.227298245250598 |
| Cu | 0.252759750536646 | 4.947890289698086 | 6.143436882545842 |
| Cu | 2.867004250950029 | 1.951699311531667 | 2.096321626389977 |
| Cu | 1.583254909810030 | 4.175218394569270 | 2.096321626389977 |
| Cu | 0.299544080380016 | 6.398670773368493 | 2.096321626389977 |
| Cu | 4.147420275495890 | 1.217609139690271 | 4.145020194994774 |
| Cu | 1.597657454206527 | 5.641136513294335 | 4.151001937255358 |
| Cu | 2.863535659533402 | 3.416973743516463 | 4.170304334684094 |
| Cu | 5.434425909810030 | 0.469397725665535 | 0.000000000000000 |
| Cu | 4.150715080380016 | 2.692850104464757 | 0.000000000000000 |
| Cu | 2.866965739240017 | 4.916369187502360 | 0.000000000000000 |
| Cu | 5.459234545395628 | 0.446154846249884 | 6.161116883395350 |
| Cu | 4.266941344474064 | 2.672193522874597 | 6.289536351976644 |
| Cu | 5.434425909810003 | 1.951699311531667 | 2.096321626389977 |
| Cu | 4.150676568670003 | 4.175218394569270 | 2.096321626389977 |
| Cu | 2.866965739239989 | 6.398670773368493 | 2.096321626389977 |
| Cu | 5.404356916887667 | 3.423936463960740 | 4.133430803076330 |
| Cu | 4.125411052707696 | 5.645222028507908 | 4.145766119216394 |
| Cu | 6.716211117710751 | 1.211875977237142 | 4.121900282924261 |
| H | 4.551944865573641 | 3.924612601298688 | 11.150386130793123 |
| H | 3.968416371278370 | 5.635591152503906 | 11.067848407118378 |
| H | -2.228755426524962 | 5.082723997924042 | 10.413034652533710 |
| H | 7.149587273918574 | 0.656375147353732 | 10.347541565251333 |
| H | -1.536016850645917 | 5.115932893983330 | 8.321748266455034 |
| H | -0.409973288962040 | 1.940199617431500 | 8.507553795954513 |
| H | -0.269734899510017 | 5.215845766688051 | 9.260047414183211 |
| H | 0.887187157147945 | 1.906892660870589 | 9.360237220388933 |
| H | 5.030250297906161 | 1.819760622527339 | 11.247666120506258 |
| H | 1.929894906859138 | 4.566749206661440 | 8.688989738447489 |
| H | 1.398966938235034 | 5.970363982949629 | 8.339843829206366 |
| H | 3.756711099416927 | 2.066385186722965 | 10.339817681540595 |
| H | 2.321474207139382 | 6.074080771053832 | 10.271327766655709 |
| H | 2.675001228460092 | 0.463737446285121 | 8.974642221120751 |
| O | 4.813411753663750 | 4.923610275415554 | 11.169274901541272 |
| O | 6.813800339178525 | -0.129357393205371 | 10.888850349329093 |
| O | -1.248808804028450 | 5.457852961696537 | 9.210681383764586 |

| | | | |
|---|---|---|---|
| O | -0.094634566632895 | 1.959841660471735 | 9.443184710147113 |
| O | 2.707504526145024 | 1.468696990217144 | 9.042485586276468 |
| O | 4.267964772107936 | 2.413693413601457 | 11.116585937032330 |
| O | 1.392550596987914 | 5.338072325661961 | 9.107283082047596 |

Cu111_CH2CO
60

| | | | |
|---|---|---|---|
| H | 3.411575549359690 | 0.962926397905455 | 8.809079903915432 |
| H | 2.111496467398592 | 1.701666546663019 | 9.724228953095134 |
| H | 2.757229589116545 | 0.092405848841241 | 10.068534501375503 |
| H | 2.196399270563117 | 3.591986622162667 | 9.978894023299480 |
| H | 3.088297402476830 | -1.696962831005828 | 10.458752000499267 |
| H | 2.818972917283224 | 5.006953198965959 | 8.373520482933511 |
| H | 4.123978464515343 | -0.705896652595795 | 11.119394434018393 |
| H | 4.149986811570198 | -2.928464340630634 | 9.206256984863789 |
| H | 6.008455422422394 | -2.237149689087981 | 8.426890918556786 |
| H | 5.523699924579118 | 0.309766897823966 | 9.797565168684512 |
| H | 5.933424923595112 | 0.747904762783419 | 11.266597198125758 |
| H | 6.084226990986465 | -3.648556210439837 | 9.049432243913998 |
| H | 0.739983929782037 | 3.047605035071033 | 10.453606225231791 |
| H | 0.831820589952318 | -0.751798456968853 | 8.257509203343167 |
| H | 0.640125397178939 | 5.433946488634763 | 9.301897130070881 |
| C | 5.701483915431098 | 1.904573610596659 | 7.890305279083883 |
| C | 0.271499501344971 | 6.034501996783823 | 8.464839167302589 |
| O | 2.524575738454000 | 0.789817015885201 | 9.275557234255718 |
| O | 1.704295696937124 | 2.819700901929357 | 10.378795433397896 |
| O | 3.148842427018590 | -0.871451928408795 | 11.020643337406023 |
| O | 3.137731822767147 | 4.788091168521864 | 9.282842608990826 |
| O | 5.761621840969838 | -2.737043632604819 | 9.242556920556213 |
| O | 5.788660869836480 | -0.043416564712166 | 10.716845234468092 |
| O | 4.981077260715763 | 0.877953385460307 | 8.366027023415045 |
| Cu | 0.494078305679993 | 0.256757570569980 | 0.000000000000000 |
| Cu | 0.494078305679993 | 2.824179229430008 | 0.000000000000000 |
| Cu | 0.494078305679993 | 5.391677911710007 | 0.000000000000000 |
| Cu | 0.542072530375329 | 5.366137892736715 | 6.292497028365766 |
| Cu | 0.495487507723906 | 0.307157178375730 | 6.104262439033591 |
| Cu | 0.490519005857311 | 2.798709595947102 | 6.176520165654401 |
| Cu | 1.235229116320017 | 1.540468399999967 | 2.096321626389977 |
| Cu | 1.235229116320017 | 4.107967082279965 | 2.096321626389977 |
| Cu | 1.235229116320017 | 6.675388741139993 | 2.096321626389977 |
| Cu | 1.971370990522500 | 2.822034779106673 | 4.115600210984290 |
| Cu | 1.976197101329859 | 0.242012205439262 | 4.118194888931489 |
| Cu | 1.943940005993361 | 5.380537805088026 | 4.141169652350754 |
| Cu | 2.717597441839991 | -1.026991770570019 | 0.000000000000000 |
| Cu | 2.717597441839991 | 1.540429888290010 | 0.000000000000000 |
| Cu | 2.717597441839991 | 4.107928570570007 | 0.000000000000000 |
| Cu | 2.674034412085467 | 1.537093726761114 | 6.139936667644832 |
| Cu | 2.708996986192001 | -1.045105614094478 | 6.147191441014720 |
| Cu | 2.715043107676272 | 4.076431875641326 | 6.142344785754495 |

| | | | |
|---|---|---|---|
| Cu | 3.458748252480015 | 0.256719058859968 | 2.096321626389977 |
| Cu | 3.458748252480015 | 2.824217741139966 | 2.096321626389977 |
| Cu | 3.458748252480015 | 5.391639399999995 | 2.096321626389977 |
| Cu | 4.205230981630715 | -1.012511072164216 | 4.137633878811767 |
| Cu | 4.210566381380301 | 4.072423700369008 | 4.138434274965289 |
| Cu | 4.207011690969678 | 1.535693362184975 | 4.166915927463804 |
| Cu | 4.941049873760016 | -2.310702600000033 | 0.000000000000000 |
| Cu | 4.941049873760016 | 0.256719058859995 | 0.000000000000000 |
| Cu | 4.941049873760016 | 2.824217741139993 | 0.000000000000000 |
| Cu | 4.925195617127700 | -2.326516399433763 | 6.146622517251764 |
| Cu | 4.890442815356566 | 0.187092669084083 | 6.260591532903562 |
| Cu | 5.682200684399993 | -1.026991770570018 | 2.096321626389977 |
| Cu | 5.682200684399993 | 1.540506911709979 | 2.096321626389977 |
| Cu | 5.682200684399993 | 4.107928570570007 | 2.096321626389977 |
| Cu | 6.410031914856929 | 0.268678982087461 | 4.183792191779823 |
| Cu | 6.418192843746751 | 2.804205984419655 | 4.157942526852185 |
| Cu | 6.407604352641522 | -2.309936142502239 | 4.109784540805592 |
| Cu | 4.938161825677263 | 2.846356376582358 | 6.300404456060084 |

Cu111_CHCOH
60

| | | | |
|---|---|---|---|
| H | 1.061235887799370 | 4.469601519990899 | 8.335782280888472 |
| H | 0.565392477057624 | 5.129042835962162 | 9.754409201128857 |
| H | 2.246690358684923 | 3.756351839279799 | 10.000593546862625 |
| H | 0.243074126207382 | 6.484299011542205 | 11.420113744707143 |
| H | 3.201998986149504 | 2.392160781539117 | 10.604387089536324 |
| H | 2.926929591915352 | 0.481889070689057 | 10.103217573204088 |
| H | 3.919991161616341 | 3.812125830967802 | 9.947465246644164 |
| H | 4.352378320218064 | 0.810737792897034 | 10.662048565846479 |
| H | 4.985915186267988 | -0.672456493348064 | 8.353726536502489 |
| H | 5.190847620581268 | 4.027567119238737 | 8.296310081828098 |
| H | 5.290253001278432 | -2.441719446171861 | 9.312380934862755 |
| H | 6.167359754952273 | -0.445976324719655 | 9.336261235837345 |
| H | 6.104352900172634 | 3.092815208199422 | 10.150394193662034 |
| H | 2.571108925151253 | -1.283481118922894 | 8.797233764768846 |
| H | 1.032479130629318 | 1.854211414207892 | 8.642150766211188 |
| C | 1.314226456928095 | 1.416942672849935 | 7.676799277434402 |
| C | 1.931684127891768 | 0.179270745477853 | 7.693611626614067 |
| O | 2.134461102474155 | -0.422928990013237 | 8.965388007696044 |
| O | 0.977371691248283 | 4.308519477022736 | 9.318451042681264 |
| O | -0.285359558623780 | 6.142919019488182 | 10.678320206354822 |
| O | 3.102480914004485 | 3.469836205487167 | 10.490222385332759 |
| O | 3.407587451403599 | 1.034692440639617 | 10.789817576129586 |
| O | 5.248080530278645 | -0.834317601187797 | 9.300886363838694 |
| O | 5.188475563129972 | 4.245951705331391 | 9.271089939648322 |
| Cu | 0.494078305679993 | 0.256757570569980 | 0.000000000000000 |
| Cu | 0.494078305679993 | 2.824179229430008 | 0.000000000000000 |
| Cu | 0.494078305679993 | 5.391677911710007 | 0.000000000000000 |
| Cu | 0.492381313485082 | 5.334242590945789 | 6.158733694954059 |

| | | | |
|---|---|---|---|
| Cu | 0.360532035935900 | 0.177644764234838 | 6.266651431751346 |
| Cu | 0.547084387432075 | 2.750218466880434 | 6.274432698876323 |
| Cu | 1.235229116320017 | 1.540468399999967 | 2.096321626389977 |
| Cu | 1.235229116320017 | 4.107967082279965 | 2.096321626389977 |
| Cu | 1.235229116320017 | 6.675388741139993 | 2.096321626389977 |
| Cu | 1.994769943997174 | 2.807631893483855 | 4.154819671461022 |
| Cu | 1.970487978832993 | 0.266517268603480 | 4.150572749691138 |
| Cu | 1.989420985990730 | 5.398536410770091 | 4.146271276080877 |
| Cu | 2.717597441839991 | -1.026991770570019 | 0.000000000000000 |
| Cu | 2.717597441839991 | 1.540429888290010 | 0.000000000000000 |
| Cu | 2.717597441839991 | 4.107928570570007 | 0.000000000000000 |
| Cu | 2.858490811571043 | 1.610159526103774 | 6.306669832981963 |
| Cu | 2.699818000409952 | -0.979799853789514 | 6.237806294228587 |
| Cu | 2.740380869212688 | 4.147156310231019 | 6.191616591021029 |
| Cu | 3.458748252480015 | 0.256719058859968 | 2.096321626389977 |
| Cu | 3.458748252480015 | 2.824217741139966 | 2.096321626389977 |
| Cu | 3.458748252480015 | 5.391639399999995 | 2.096321626389977 |
| Cu | 4.184491791664592 | -1.026876373082894 | 4.138485256131592 |
| Cu | 4.199035932603561 | 4.102081218444444 | 4.121497219012407 |
| Cu | 4.180169179351410 | 1.526599629830722 | 4.139879173412684 |
| Cu | 4.941049873760016 | -2.310702600000033 | 0.000000000000000 |
| Cu | 4.941049873760016 | 0.256719058859995 | 0.000000000000000 |
| Cu | 4.941049873760016 | 2.824217741139993 | 0.000000000000000 |
| Cu | 4.898129844061875 | -2.300886530502078 | 6.151996326627711 |
| Cu | 5.001493396607266 | 0.237125866817266 | 6.150197584185888 |
| Cu | 5.682200684399993 | -1.026991770570018 | 2.096321626389977 |
| Cu | 5.682200684399993 | 1.540506911709979 | 2.096321626389977 |
| Cu | 5.682200684399993 | 4.107928570570007 | 2.096321626389977 |
| Cu | 6.455293630706188 | 0.208759776511327 | 4.139598817879657 |
| Cu | 6.442640626490968 | 2.828563850097792 | 4.132767635776768 |
| Cu | 6.431657892250536 | -2.329622577166210 | 4.144143378008446 |
| Cu | 4.945191689366456 | 2.813511845718013 | 6.143372750495874 |

Cu111-HOCCOH
61

| | | | |
|---|---|---|---|
| C | 2.632766476702409 | 2.698921198166845 | 7.837010352147724 |
| C | 3.492485367785515 | 3.779632257071055 | 7.772026469304732 |
| Cu | 0.299505568670004 | 0.469397725665535 | 0.000000000000000 |
| Cu | -0.984205260760010 | 2.692850104464757 | 0.000000000000000 |
| Cu | -2.267954601900009 | 4.916369187502360 | 0.000000000000000 |
| Cu | -2.252977081987412 | 4.906925973244236 | 6.181107037843484 |
| Cu | 0.275061042229680 | 0.459080817588963 | 6.172605372263320 |
| Cu | -0.989836925452653 | 2.678359881534728 | 6.162263567825236 |
| Cu | 0.299505568670032 | 1.951699311531667 | 2.096321626389977 |
| Cu | -0.984243772469967 | 4.175218394569270 | 2.096321626389977 |
| Cu | -2.267954601899981 | 6.398670773368493 | 2.096321626389977 |
| Cu | 0.321911105111760 | 3.413176040383029 | 4.147442580194780 |
| Cu | 1.579123373623825 | 1.205830765448242 | 4.135619375315979 |
| Cu | -0.993502983280949 | 5.647382373652380 | 4.126803198553246 |

| | | | |
|---|---|---|---|
| Cu | 2.867004250950002 | 0.469397725665535 | 0.000000000000000 |
| Cu | 1.583293421519988 | 2.692850104464757 | 0.000000000000000 |
| Cu | 0.299544080379989 | 4.916369187502360 | 0.000000000000000 |
| Cu | 1.498365081747322 | 2.636384808347387 | 6.252981581063274 |
| Cu | 2.858270384500549 | 0.424424087545526 | 6.166148644625744 |
| Cu | 0.276960515412790 | 4.914657408803083 | 6.170365650680427 |
| Cu | 2.867004250950029 | 1.951699311531667 | 2.096321626389977 |
| Cu | 1.583254909810030 | 4.175218394569270 | 2.096321626389977 |
| Cu | 0.299544080380016 | 6.398670773368493 | 2.096321626389977 |
| Cu | 4.150610111748046 | 1.214333332497612 | 4.136354266787226 |
| Cu | 1.611919515384359 | 5.626938814852197 | 4.161482004874659 |
| Cu | 2.861878900214673 | 3.426853320101269 | 4.173995443099976 |
| Cu | 5.434425909810030 | 0.469397725665535 | 0.000000000000000 |
| Cu | 4.150715080380016 | 2.692850104464757 | 0.000000000000000 |
| Cu | 2.866965739240017 | 4.916369187502360 | 0.000000000000000 |
| Cu | 5.437701784213403 | 0.448513924714478 | 6.173121022360257 |
| Cu | 4.213567728596574 | 2.634756389542598 | 6.219683858913061 |
| Cu | 5.434425909810003 | 1.951699311531667 | 2.096321626389977 |
| Cu | 4.150676568670003 | 4.175218394569270 | 2.096321626389977 |
| Cu | 2.866965739239989 | 6.398670773368493 | 2.096321626389977 |
| Cu | 5.423480960575162 | 3.418692907440116 | 4.125917130094128 |
| Cu | 4.144402765883274 | 5.643944127623687 | 4.141903631455797 |
| Cu | 6.720648538121345 | 1.198378320418592 | 4.125690667156114 |
| Cu | 2.879819992396576 | 4.971278590431054 | 6.313298734075221 |
| H | 0.288062100065773 | 4.196935103983975 | 8.545036564213884 |
| H | -0.477065132738014 | 5.163343794203964 | 10.379259883455232 |
| H | 1.054994185056407 | 4.003420308439036 | 9.952465285560972 |
| H | 3.705018690214292 | 0.114597832198443 | 10.951373134446234 |
| H | 2.477100843288677 | 3.804707028336280 | 11.676874515936534 |
| H | 3.715469694498367 | 1.688104906949969 | 10.106361334134027 |
| H | 3.161434342782265 | 4.169474438306721 | 10.317436620025976 |
| H | 5.214100918971023 | 1.279181716485398 | 10.376328152268174 |
| H | 6.446398564716708 | 1.452653489154945 | 8.369413321196447 |
| H | -3.087149088617508 | 5.724067822798726 | 9.116154435871037 |
| H | 0.126384411714860 | 0.446390125306230 | 9.612264964411327 |
| H | -0.609892347183424 | 2.460681676562379 | 9.415891538694815 |
| H | -1.722252475376754 | 6.194165847262711 | 10.585382341182621 |
| H | 1.945461450236807 | 1.163381036735135 | 8.885352300145424 |
| H | 5.108030047233085 | 3.528586191572360 | 8.908312049665259 |
| O | 0.140982442126917 | 4.001461636079633 | 9.507807900355909 |
| O | -0.839836622093894 | 5.894094865921867 | 11.001188854503912 |
| O | 2.366473654214739 | 4.355867719497478 | 10.883029500066284 |
| O | 4.315431209441143 | 1.285818349411550 | 10.810035430686694 |
| O | 6.581012607820910 | 1.598774908845333 | 9.348825202013742 |
| O | -2.871201222519016 | 6.600903076153473 | 9.545648009184555 |
| O | 2.555206306816027 | 1.948910049889877 | 8.991949342117634 |
| O | 4.297559912550654 | 4.078923608870856 | 8.964583886052784 |

Cu111-OCCH
61

| | | | |
|---|---|---|---|
| C | 2.632766476702409 | 2.698921198166845 | 7.837010352147724 |
| C | 3.492485367785515 | 3.779632257071055 | 7.772026469304732 |
| Cu | 0.299505568670004 | 0.469397725665535 | 0.000000000000000 |
| Cu | -0.984205260760010 | 2.692850104464757 | 0.000000000000000 |
| Cu | -2.267954601900009 | 4.916369187502360 | 0.000000000000000 |
| Cu | -2.252977081987412 | 4.906925973244236 | 6.181107037843484 |
| Cu | 0.275061042229680 | 0.459080817588963 | 6.172605372263320 |
| Cu | -0.989836925452653 | 2.678359881534728 | 6.162263567825236 |
| Cu | 0.299505568670032 | 1.951699311531667 | 2.096321626389977 |
| Cu | -0.984243772469967 | 4.175218394569270 | 2.096321626389977 |
| Cu | -2.267954601899981 | 6.398670773368493 | 2.096321626389977 |
| Cu | 0.321911105111760 | 3.413176040383029 | 4.147442580194780 |
| Cu | 1.579123373623825 | 1.205830765448242 | 4.135619375315979 |
| Cu | -0.993502983280949 | 5.647382373652380 | 4.126803198553246 |
| Cu | 2.867004250950002 | 0.469397725665535 | 0.000000000000000 |
| Cu | 1.583293421519988 | 2.692850104464757 | 0.000000000000000 |
| Cu | 0.299544080379989 | 4.916369187502360 | 0.000000000000000 |
| Cu | 1.498365081747322 | 2.636384808347387 | 6.252981581063274 |
| Cu | 2.858270384500549 | 0.424424087545526 | 6.166148644625744 |
| Cu | 0.276960515412790 | 4.914657408803083 | 6.170365650680427 |
| Cu | 2.867004250950029 | 1.951699311531667 | 2.096321626389977 |
| Cu | 1.583254909810030 | 4.175218394569270 | 2.096321626389977 |
| Cu | 0.299544080380016 | 6.398670773368493 | 2.096321626389977 |
| Cu | 4.150610111748046 | 1.214333332497612 | 4.136354266787226 |
| Cu | 1.611919515384359 | 5.626938814852197 | 4.161482004874659 |
| Cu | 2.861878900214673 | 3.426853320101269 | 4.173995443099976 |
| Cu | 5.434425909810030 | 0.469397725665535 | 0.000000000000000 |
| Cu | 4.150715080380016 | 2.692850104464757 | 0.000000000000000 |
| Cu | 2.866965739240017 | 4.916369187502360 | 0.000000000000000 |
| Cu | 5.437701784213403 | 0.448513924714478 | 6.173121022360257 |
| Cu | 4.213567728596574 | 2.634756389542598 | 6.219683858913061 |
| Cu | 5.434425909810003 | 1.951699311531667 | 2.096321626389977 |
| Cu | 4.150676568670003 | 4.175218394569270 | 2.096321626389977 |
| Cu | 2.866965739239989 | 6.398670773368493 | 2.096321626389977 |
| Cu | 5.423480960575162 | 3.418692907440116 | 4.125917130094128 |
| Cu | 4.144402765883274 | 5.643944127623687 | 4.141903631455797 |
| Cu | 6.720648538121345 | 1.198378320418592 | 4.125690667156114 |
| Cu | 2.879819992396576 | 4.971278590431054 | 6.313298734075221 |
| H | 0.288062100065773 | 4.196935103983975 | 8.545036564213884 |
| H | -0.477065132738014 | 5.163343794203964 | 10.379259883455232 |
| H | 1.054994185056407 | 4.003420308439036 | 9.952465285560972 |
| H | 3.705018690214292 | 0.114597832198443 | 10.951373134446234 |
| H | 2.477100843288677 | 3.804707028336280 | 11.676874515936534 |
| H | 3.715469694498367 | 1.688104906949969 | 10.106361334134027 |
| H | 3.161434342782265 | 4.169474438306721 | 10.317436620025976 |
| H | 5.214100918971023 | 1.279181716485398 | 10.376328152268174 |
| H | 6.446398564716708 | 1.452653489154945 | 8.369413321196447 |
| H | -3.087149088617508 | 5.724067822798726 | 9.116154435871037 |
| H | 0.126384411714860 | 0.446390125306230 | 9.612264964411327 |

| | | | |
|---|---|---|---|
| H | -0.609892347183424 | 2.460681676562379 | 9.415891538694815 |
| H | -1.722252475376754 | 6.194165847262711 | 10.585382341182621 |
| H | 1.945461450236807 | 1.163381036735135 | 8.885352300145424 |
| H | 5.108030047233085 | 3.528586191572360 | 8.908312049665259 |
| O | 0.140982442126917 | 4.001461636079633 | 9.507807900355909 |
| O | -0.839836622093894 | 5.894094865921867 | 11.001188854503912 |
| O | 2.366473654214739 | 4.355867719497478 | 10.883029500066284 |
| O | 4.315431209441143 | 1.285818349411550 | 10.810035430686694 |
| O | 6.581012607820910 | 1.598774908845333 | 9.348825202013742 |
| O | -2.871201222519016 | 6.600903076153473 | 9.545648009184555 |
| O | 2.555206306816027 | 1.948910049889877 | 8.991949342117634 |
| O | 4.297559912550654 | 4.078923608870856 | 8.964583886052784 |

Cu111_OCCOH

60

| | | | |
|---|---|---|---|
| C | 2.659894104032861 | 2.080714982911912 | 7.679222398918974 |
| C | 3.231114169610874 | 3.378534834777698 | 7.787985334874652 |
| Cu | 0.299505568670004 | 0.469397725665535 | 0.000000000000000 |
| Cu | -0.984205260760010 | 2.692850104464757 | 0.000000000000000 |
| Cu | -2.267954601900009 | 4.916369187502360 | 0.000000000000000 |
| Cu | -2.215009946254366 | 4.964678134468858 | 6.168948093595368 |
| Cu | 0.256901327640654 | 0.435708928918047 | 6.170773093548606 |
| Cu | -0.979081894035908 | 2.700353238450000 | 6.170402599923103 |
| Cu | 0.299505568670032 | 1.951699311531667 | 2.096321626389977 |
| Cu | -0.984243772469967 | 4.175218394569270 | 2.096321626389977 |
| Cu | -2.267954601899981 | 6.398670773368493 | 2.096321626389977 |
| Cu | 0.317087194477439 | 3.445958543910317 | 4.135527170227385 |
| Cu | 1.578924104183777 | 1.230263001617610 | 4.134943874528626 |
| Cu | -0.974561248482352 | 5.712667051374767 | 4.150882278299926 |
| Cu | 2.867004250950002 | 0.469397725665535 | 0.000000000000000 |
| Cu | 1.583293421519988 | 2.692850104464757 | 0.000000000000000 |
| Cu | 0.299544080379989 | 4.916369187502360 | 0.000000000000000 |
| Cu | 1.430575668116682 | 2.693715892967295 | 6.237473223304729 |
| Cu | 2.873295013066346 | 0.571226252095535 | 6.232459985774371 |
| Cu | 0.287390798918838 | 4.956963798637942 | 6.182748342236047 |
| Cu | 2.867004250950029 | 1.951699311531667 | 2.096321626389977 |
| Cu | 1.583254909810030 | 4.175218394569270 | 2.096321626389977 |
| Cu | 0.299544080380016 | 6.398670773368493 | 2.096321626389977 |
| Cu | 4.167523754119618 | 1.235112381701020 | 4.132103316945916 |
| Cu | 1.610521909446077 | 5.647091047776153 | 4.154570448544205 |
| Cu | 2.870063702936063 | 3.423329659105439 | 4.155924898844654 |
| Cu | 5.434425909810030 | 0.469397725665535 | 0.000000000000000 |
| Cu | 4.150715080380016 | 2.692850104464757 | 0.000000000000000 |
| Cu | 2.866965739240017 | 4.916369187502360 | 0.000000000000000 |
| Cu | 5.464910838821358 | 0.435768002797641 | 6.163729229960426 |
| Cu | 4.308894167105590 | 2.697903524090342 | 6.216498079070611 |
| Cu | 5.434425909810003 | 1.951699311531667 | 2.096321626389977 |
| Cu | 4.150676568670003 | 4.175218394569270 | 2.096321626389977 |
| Cu | 2.866965739239989 | 6.398670773368493 | 2.096321626389977 |

| | | | |
|---|---|---|---|
| Cu | 5.439367405216758 | 3.447589767669013 | 4.120604166380134 |
| Cu | 4.151743943708643 | 5.660412114562363 | 4.150190084577877 |
| Cu | 6.723071240696532 | 1.221319464770887 | 4.133040336102945 |
| Cu | 2.888318578631669 | 4.826057978990486 | 6.288599729588275 |
| H | 0.320745182307357 | 3.708824768609521 | 8.791296093085609 |
| H | -0.631742446550545 | 4.708060409541997 | 10.451284306369171 |
| H | 0.920260997563940 | 3.425570564784236 | 10.282191294557153 |
| H | -0.417975623558843 | 6.323142188573835 | 10.987176749944584 |
| H | 2.530720838944574 | 2.779741604338482 | 11.622104587949423 |
| H | 3.584446434110920 | 1.270998636661733 | 10.100437423329931 |
| H | 2.814901341100593 | 3.651855569119257 | 10.337802235751617 |
| H | 5.043193738099363 | 0.930130448294619 | 10.541043618354401 |
| H | 6.235929349328501 | 1.117853199234959 | 8.554688974350103 |
| H | 4.270208625635084 | 5.239087938506401 | 9.081654807595148 |
| H | 0.080099245469917 | 0.056680538610580 | 9.594839161388403 |
| H | -0.798212335529136 | 2.069997628142186 | 9.613515982112633 |
| H | -1.930916212616222 | 5.744872357640042 | 10.438173030120131 |
| H | 1.889570466293842 | 0.601218953759415 | 8.780869774942042 |
| O | 0.053156982387574 | 3.530408421384045 | 9.722245013813898 |
| O | -1.079314397670443 | 5.476111732924809 | 10.976177313383722 |
| O | 2.196798385461878 | 3.552204497054519 | 11.131378674800528 |
| O | 4.106568627766644 | 0.919909459650603 | 10.879252452904844 |
| O | 6.478914438750459 | 1.165201393989582 | 9.511468429201983 |
| O | -2.996592230951370 | 6.116225219787409 | 9.419135284029345 |
| O | 2.464665734760165 | 1.417564295984495 | 8.882155142022668 |
| O | 3.523147017966722 | 3.955223069994116 | 8.91497112538400З |

Cu111-Cd
55

| | | | |
|---|---|---|---|
| H | 2.506256860635178 | 3.193834384761062 | 8.423212404792242 |
| H | 2.495747131026516 | 4.516062100874660 | 9.936752848968519 |
| H | 3.302832230247625 | 2.598847942246343 | 9.696991198443950 |
| H | 3.329970715634251 | -1.687658861562995 | 9.922978603922012 |
| H | 4.538303138399544 | 0.862682618595917 | 9.867115329426491 |
| H | 4.323656350262135 | -0.802842528094437 | 8.362330560450056 |
| H | 5.423746311953199 | 2.175181758853816 | 9.915867396853779 |
| H | 5.362677923298143 | -1.323339143417437 | 9.433134512085607 |
| H | 0.138490896344265 | 1.625381821592472 | 8.300125223301466 |
| H | 0.467032177892512 | 6.542019984519178 | 8.458012178684621 |
| H | 0.365421585115960 | -0.009754539742706 | 9.523886399818352 |
| H | 0.988658639427755 | 2.203390362955298 | 9.483042657595499 |
| H | 1.653234472159625 | 6.001763033465907 | 10.000199943066026 |
| O | 2.457905828526315 | 3.136595931618701 | 9.418112749506724 |
| O | 2.509304341994726 | 5.504644224530585 | 10.309888910458113 |
| O | 4.532808236478015 | 1.813418976132931 | 10.145319945105943 |
| O | 4.469689696340176 | -0.864547941706318 | 9.349346523028535 |
| O | 0.159119393798147 | 1.669340244048924 | 9.301064566025850 |
| O | 0.407235222496917 | 6.688897871792253 | 9.443434639148562 |
| Cu | 0.494078305679993 | 0.256757570569980 | 0.000000000000000 |

| | | | |
|---|---|---|---|
| Cu | 0.494078305679993 | 2.824179229430008 | 0.000000000000000 |
| Cu | 0.494078305679993 | 5.391677911710007 | 0.000000000000000 |
| Cu | 0.530542688008533 | 5.372527630656516 | 6.199080412629060 |
| Cu | 0.527065460213048 | 0.275781141547112 | 6.188156351050212 |
| Cu | 0.496005716894043 | 2.832528355055265 | 6.190331424506256 |
| Cu | 1.235229116320017 | 1.540468399999967 | 2.096321626389977 |
| Cu | 1.235229116320017 | 4.107967082279965 | 2.096321626389977 |
| Cu | 1.235229116320017 | 6.675388741139993 | 2.096321626389977 |
| Cu | 1.982606097915862 | 2.825037124357377 | 4.141081643132342 |
| Cu | 1.977751770268750 | 0.258664495837127 | 4.136000377851224 |
| Cu | 1.976358220683216 | 5.396903529327536 | 4.133057764695956 |
| Cu | 2.717597441839991 | -1.026991770570019 | 0.000000000000000 |
| Cu | 2.717597441839991 | 1.540429888290010 | 0.000000000000000 |
| Cu | 2.717597441839991 | 4.107928570570007 | 0.000000000000000 |
| Cu | 2.668097163551046 | 1.518829884283892 | 6.216724219625607 |
| Cu | 2.713284775468001 | -1.023202823072643 | 6.213291860881404 |
| Cu | 2.677441720755833 | 4.141877312687303 | 6.191683763104923 |
| Cu | 3.458748252480015 | 0.256719058859968 | 2.096321626389977 |
| Cu | 3.458748252480015 | 2.824217741139966 | 2.096321626389977 |
| Cu | 3.458748252480015 | 5.391639399999995 | 2.096321626389977 |
| Cu | 4.195329818016676 | -1.027362693730396 | 4.134569871789154 |
| Cu | 4.203203021477082 | 4.107913540234774 | 4.149395784787166 |
| Cu | 4.198431053233976 | 1.546530314971791 | 4.153511299066760 |
| Cu | 4.941049873760016 | -2.310702600000033 | 0.000000000000000 |
| Cu | 4.941049873760016 | 0.256719058859995 | 0.000000000000000 |
| Cu | 4.941049873760016 | 2.824217741139993 | 0.000000000000000 |
| Cu | 4.941554907505323 | -2.260530821270728 | 6.201292876163459 |
| Cu | 4.938715705636335 | 0.214464490230500 | 6.188542494097336 |
| Cu | 5.682200684399993 | -1.026991770570018 | 2.096321626389977 |
| Cu | 5.682200684399993 | 1.540506911709979 | 2.096321626389977 |
| Cu | 5.682200684399993 | 4.107928570570007 | 2.096321626389977 |
| Cu | 6.420493154910885 | 0.264433246821336 | 4.138573137629412 |
| Cu | 6.416496372962226 | 2.824810753288340 | 4.145826051146503 |
| Cu | 6.419609659059180 | -2.313558654562228 | 4.139344762767497 |
| Cd | 4.928865563030136 | 2.825834848538662 | 6.674836875057070 |

Cu111-Cd_H
56

| | | | |
|---|---|---|---|
| H | 1.906424044256707 | 5.031045049219098 | 8.415051315534706 |
| H | 1.763499245106430 | 6.268875036839278 | 9.967226398738839 |
| H | 2.631797159701817 | 4.364589750797856 | 9.704369635466479 |
| H | 2.585483047683913 | 0.051599224418795 | 10.022444350897956 |
| H | 3.820804614001401 | 2.599579317561432 | 9.945746579580653 |
| H | 3.618479275543490 | 0.824753113296513 | 8.457079030718621 |
| H | 4.701230556931749 | 3.920369093178582 | 9.974203891123015 |
| H | 4.672439808967841 | 0.382757920982996 | 9.535417760657957 |
| H | 6.108529347592228 | -0.427852942253319 | 8.312070010394921 |
| H | 6.351893575782910 | -3.212612875891645 | 8.485720906545652 |
| H | 6.318722921454133 | -2.072198492991613 | 9.560467653599460 |

| | | | |
|---|---|---|---|
| H | 0.337493707776214 | 3.992994400336494 | 9.460676144386293 |
| H | 0.903466636462460 | 0.057175324513483 | 10.022058645242870 |
| H | 2.997914652936631 | 2.908394218513524 | 7.306846844981157 |
| O | 1.810428624743741 | 4.926235440848679 | 9.401463733369534 |
| O | 1.742578987911682 | -0.448749992890018 | 10.362743314349903 |
| O | 3.816275423330453 | 3.550605633058124 | 10.216310032445366 |
| O | 3.770617856271584 | 0.821288802287740 | 9.442586817828612 |
| O | 6.174346615066933 | -0.396479734959060 | 9.311578977766182 |
| O | 6.331659628697057 | -3.076407105866875 | 9.474867927549147 |
| Cu | 0.494078305679993 | 0.256757570569980 | 0.000000000000000 |
| Cu | 0.494078305679993 | 2.824179229430008 | 0.000000000000000 |
| Cu | 0.494078305679993 | 5.391677911710007 | 0.000000000000000 |
| Cu | 0.565025195806909 | 5.385094803971795 | 6.194579454572188 |
| Cu | 0.572327824663111 | 0.298880747639098 | 6.203106853077557 |
| Cu | 0.505352340428822 | 2.843174240123838 | 6.183155882119877 |
| Cu | 1.235229116320017 | 1.540468399999967 | 2.096321626389977 |
| Cu | 1.235229116320017 | 4.107967082279965 | 2.096321626389977 |
| Cu | 1.235229116320017 | 6.675388741139993 | 2.096321626389977 |
| Cu | 1.998602534394896 | 2.834291064084059 | 4.133093398774649 |
| Cu | 2.004682627516332 | 0.282102308111027 | 4.157894412385610 |
| Cu | 2.003803338811422 | 5.395997766059035 | 4.151697782346528 |
| Cu | 2.717597441839991 | -1.026991770570019 | 0.000000000000000 |
| Cu | 2.717597441839991 | 1.540429888290010 | 0.000000000000000 |
| Cu | 2.717597441839991 | 4.107928570570007 | 0.000000000000000 |
| Cu | 2.708735575793554 | 1.561053265877351 | 6.258634632660860 |
| Cu | 2.753642022626917 | -0.998753703359622 | 6.224950040049172 |
| Cu | 2.714337432382284 | 4.159992295397039 | 6.228408093659866 |
| Cu | 3.458748252480015 | 0.256719058859968 | 2.096321626389977 |
| Cu | 3.458748252480015 | 2.824217741139966 | 2.096321626389977 |
| Cu | 3.458748252480015 | 5.391639399999995 | 2.096321626389977 |
| Cu | 4.214273179792536 | -1.022604255793960 | 4.132061814083015 |
| Cu | 4.220601112576969 | 4.114064730097164 | 4.157426328644897 |
| Cu | 4.215528248189443 | 1.559331911234461 | 4.153509697917079 |
| Cu | 4.941049873760016 | -2.310702600000033 | 0.000000000000000 |
| Cu | 4.941049873760016 | 0.256719058859995 | 0.000000000000000 |
| Cu | 4.941049873760016 | 2.824217741139993 | 0.000000000000000 |
| Cu | 4.984120547140303 | -2.236650542296195 | 6.210476245747662 |
| Cu | 4.969611508244640 | 0.229365805578023 | 6.175746149154986 |
| Cu | 5.682200684399993 | -1.026991770570018 | 2.096321626389977 |
| Cu | 5.682200684399993 | 1.540506911709979 | 2.096321626389977 |
| Cu | 5.682200684399993 | 4.107928570570007 | 2.096321626389977 |
| Cu | 6.441871599306681 | 0.270635166860540 | 4.132900392885679 |
| Cu | 6.433355518681391 | 2.834078285510601 | 4.143879746289813 |
| Cu | 6.442170709347596 | -2.305474075371769 | 4.138742039172789 |
| Cd | 5.033194641458794 | 2.841322755564658 | 6.688103202592514 |

Cu111-Cd_2H
57

| | | | |
|---|---|---|---|
| H | 1.802013113455527 | 4.894601646395842 | 8.428814582646284 |

| | | | |
|---|---|---|---|
| H | 1.646222590432358 | 6.164600053457004 | 9.956516400027766 |
| H | 2.505008496816772 | 4.255221609254179 | 9.746608691117705 |
| H | 2.467296833402385 | -0.042787843138306 | 10.011184787433606 |
| H | 3.687875707336019 | 2.502150731292864 | 10.015201580264804 |
| H | 3.535333906122046 | 0.774664258875813 | 8.481330155399617 |
| H | 4.557649874919010 | 3.825696369518850 | 10.062963721690220 |
| H | 4.556648576648957 | 0.296799256191663 | 9.572514859942844 |
| H | 5.974514222355705 | -0.524271150465266 | 8.343242695591565 |
| H | 6.192705738576044 | -3.329675951907157 | 8.557391763181595 |
| H | 6.175878034974704 | -2.174295283978270 | 9.610454239412148 |
| H | 0.213803249615218 | 3.886958498833256 | 9.485877249930013 |
| H | 0.781064932146048 | -0.047218271217136 | 10.045595999352768 |
| H | 1.128652261517628 | 1.474799814833509 | 7.176825998903543 |
| H | 2.997914652936607 | 2.908394218513549 | 7.306846844981139 |
| O | 1.690294321939133 | 4.814093066126845 | 9.415423067094428 |
| O | 1.629711199511968 | -0.552044852367612 | 10.352492811875226 |
| O | 3.672309554207399 | 3.450253803989618 | 10.295672482133801 |
| O | 3.658494013813272 | 0.740392143412863 | 9.470296960774940 |
| O | 6.047725102870529 | -0.499013399917698 | 9.341878039514881 |
| O | 6.176387446218743 | -3.179886418840760 | 9.541277638221640 |
| Cu | 0.494078305679993 | 0.256757570569980 | 0.000000000000000 |
| Cu | 0.494078305679993 | 2.824179229430008 | 0.000000000000000 |
| Cu | 0.494078305679993 | 5.391677911710007 | 0.000000000000000 |
| Cu | 0.531918762431107 | 5.350841742195986 | 6.190871465645507 |
| Cu | 0.527566297752455 | 0.240171389876346 | 6.226094640065598 |
| Cu | 0.456661911458861 | 2.815675508557254 | 6.225797958514596 |
| Cu | 1.235229116320017 | 1.540468399999967 | 2.096321626389977 |
| Cu | 1.235229116320017 | 4.107967082279965 | 2.096321626389977 |
| Cu | 1.235229116320017 | 6.675388741139993 | 2.096321626389977 |
| Cu | 1.980285480214350 | 2.825127034905104 | 4.139930878696457 |
| Cu | 1.986163250594714 | 0.267869975599634 | 4.151880007858767 |
| Cu | 1.983357916773507 | 5.387330412920631 | 4.149397905363981 |
| Cu | 2.717597441839991 | -1.026991770570019 | 0.000000000000000 |
| Cu | 2.717597441839991 | 1.540429888290010 | 0.000000000000000 |
| Cu | 2.717597441839991 | 4.107928570570007 | 0.000000000000000 |
| Cu | 2.661271230774584 | 1.528203877340338 | 6.318283197802645 |
| Cu | 2.730805705271771 | -1.036228998524028 | 6.214928653905791 |
| Cu | 2.689584593968386 | 4.146144264585693 | 6.217820643589303 |
| Cu | 3.458748252480015 | 0.256719058859968 | 2.096321626389977 |
| Cu | 3.458748252480015 | 2.824217741139966 | 2.096321626389977 |
| Cu | 3.458748252480015 | 5.391639399999995 | 2.096321626389977 |
| Cu | 4.196407265699009 | -1.031542233660481 | 4.125125391216518 |
| Cu | 4.204775734751490 | 4.100538026065656 | 4.149010885751517 |
| Cu | 4.179521743904080 | 1.545844181189192 | 4.164349439720715 |
| Cu | 4.941049873760016 | -2.310702600000033 | 0.000000000000000 |
| Cu | 4.941049873760016 | 0.256719058859995 | 0.000000000000000 |
| Cu | 4.941049873760016 | 2.824217741139993 | 0.000000000000000 |
| Cu | 4.930838560470755 | -2.268459387391525 | 6.191741573922312 |
| Cu | 4.928776197607410 | 0.197064630551217 | 6.176745673568107 |
| Cu | 5.682200684399993 | -1.026991770570018 | 2.096321626389977 |

| | | | |
|---|---|---|---|
| Cu | 5.682200684399993 | 1.540506911709979 | 2.096321626389977 |
| Cu | 5.682200684399993 | 4.107928570570007 | 2.096321626389977 |
| Cu | 6.427119742519250 | 0.243933393141423 | 4.142969420238666 |
| Cu | 6.420134396622453 | 2.830587484535531 | 4.159289096167597 |
| Cu | 6.423841311530739 | -2.319071405482432 | 4.128967207543061 |
| Cd | 4.985517843599757 | 2.795500057767948 | 6.683476578704559 |

Cu111-Cd_CCO
58

| | | | |
|---|---|---|---|
| H | 0.839134022924608 | 4.106221928510250 | 10.572078730515598 |
| H | 0.910632986562956 | 5.285423889810849 | 11.723816757770408 |
| H | 2.090874162262391 | 3.585783756351213 | 11.890969962926732 |
| H | 1.553899563616496 | -0.390176515137554 | 11.823461273198818 |
| H | 2.907700745862813 | 2.060932087742046 | 11.711487409773195 |
| H | 2.417467002389459 | 0.753009940348396 | 10.103367643063926 |
| H | 3.809356974812733 | 3.459798662879813 | 11.764533856532605 |
| H | 3.614770857554121 | 0.137394425280688 | 10.989742260142394 |
| H | 5.457037903941500 | -0.927337148662506 | 10.265357665985816 |
| H | 5.194040606258337 | 3.847064571914346 | 10.166786949890403 |
| H | 5.103569180405783 | -2.620486430450855 | 11.133235609693186 |
| H | 5.617559242644373 | -0.458069116808467 | 11.739873122463651 |
| H | 6.654347190589513 | 3.338865598092247 | 11.858281365337854 |
| C | 0.733738515540149 | 1.907160742036874 | 8.668299562625105 |
| C | 1.267415329467138 | 1.498679126472480 | 7.583233099760664 |
| O | 0.951910002373045 | 4.265806785794314 | 11.532859197855849 |
| O | 0.810784279491357 | 6.733663491114610 | 12.135505732744427 |
| O | 2.982426383157332 | 2.995180343422753 | 12.144181437857201 |
| O | 2.741028392986623 | 0.657340532524297 | 11.024653305934208 |
| O | 4.933454797563731 | -0.753316257542909 | 11.100772324607645 |
| O | 5.102433066833652 | 4.089944721375247 | 11.110313984963446 |
| O | 0.238327407802086 | 2.267006021448988 | 9.747977701677168 |
| Cu | 0.494078305679993 | 0.256757570569980 | 0.000000000000000 |
| Cu | 0.494078305679993 | 2.824179229430008 | 0.000000000000000 |
| Cu | 0.494078305679993 | 5.391677911710007 | 0.000000000000000 |
| Cu | 0.509284766498654 | 5.375268583181442 | 6.223574874298816 |
| Cu | 0.521986150458761 | 0.214429606099156 | 6.276734830991524 |
| Cu | 0.457960102108841 | 2.858417213964263 | 6.275059378839640 |
| Cu | 1.235229116320017 | 1.540468399999967 | 2.096321626389977 |
| Cu | 1.235229116320017 | 4.107967082279965 | 2.096321626389977 |
| Cu | 1.235229116320017 | 6.675388741139993 | 2.096321626389977 |
| Cu | 1.976817366771839 | 2.824674105776012 | 4.169652069392258 |
| Cu | 1.968854878239204 | 0.249842358487021 | 4.153722991429158 |
| Cu | 1.959502816853214 | 5.390220819649301 | 4.162127813226665 |
| Cu | 2.717597441839991 | -1.026991770570019 | 0.000000000000000 |
| Cu | 2.717597441839991 | 1.540429888290010 | 0.000000000000000 |
| Cu | 2.717597441839991 | 4.107928570570007 | 0.000000000000000 |
| Cu | 2.715250575438296 | 1.481119857672599 | 6.287199312276905 |
| Cu | 2.716136289148315 | -1.065561961689162 | 6.231261809904194 |
| Cu | 2.680352868022319 | 4.150053931921078 | 6.232284088556483 |

| | | | |
|---|---|---|---|
| Cu | 3.458748252480015 | 0.256719058859968 | 2.096321626389977 |
| Cu | 3.458748252480015 | 2.824217741139966 | 2.096321626389977 |
| Cu | 3.458748252480015 | 5.391639399999995 | 2.096321626389977 |
| Cu | 4.189841973174454 | -1.023875167931401 | 4.161009288487045 |
| Cu | 4.193149536461564 | 4.094040994537708 | 4.176682315418258 |
| Cu | 4.163055806845310 | 1.545022218877298 | 4.192522476244336 |
| Cu | 4.941049873760016 | -2.310702600000033 | 0.000000000000000 |
| Cu | 4.941049873760016 | 0.256719058859995 | 0.000000000000000 |
| Cu | 4.941049873760016 | 2.824217741139993 | 0.000000000000000 |
| Cu | 4.884854467594383 | -2.280499304135676 | 6.226647694602719 |
| Cu | 4.915028890106294 | 0.211986276681284 | 6.222483541004164 |
| Cu | 5.682200684399993 | -1.026991770570018 | 2.096321626389977 |
| Cu | 5.682200684399993 | 1.540506911709979 | 2.096321626389977 |
| Cu | 5.682200684399993 | 4.107928570570007 | 2.096321626389977 |
| Cu | 6.417461771223640 | 0.245354161452336 | 4.162923356842638 |
| Cu | 6.416638534285490 | 2.843940738173957 | 4.183448817285412 |
| Cu | 6.405303977757220 | -2.321008859708166 | 4.173668511102635 |
| Cd | 4.923746950398594 | 2.819643455231662 | 6.778019239927835 |

Cu111-Cd_CH2CO
60

| | | | |
|---|---|---|---|
| H | 2.090456017460406 | 3.582678514299525 | 9.449502478654415 |
| H | 1.815667202541785 | 4.243606884898269 | 11.261362080689192 |
| H | 2.625866344809786 | 2.767642632897486 | 11.196799022439608 |
| H | 2.528337797383533 | 6.221824252783946 | 11.037105422623775 |
| H | 3.942513858917926 | 0.922855771851878 | 10.989886794175725 |
| H | 3.706853310515036 | -0.694060513475031 | 9.106907386717561 |
| H | 4.770070996750518 | 2.260410042116573 | 10.984247324131646 |
| H | 4.750128201876151 | -1.164220352617127 | 10.178149367523417 |
| H | 6.490642170250768 | -1.818146515173131 | 9.180070409457528 |
| H | 6.288741380167079 | 2.811369210466760 | 9.230787583797058 |
| H | 6.223716595538528 | 4.041479461929727 | 10.217807165767518 |
| H | 0.348233639459012 | 2.413051214826222 | 10.588652481384583 |
| H | 0.962802293593067 | 6.197611844925851 | 11.117408972029562 |
| H | 2.719842380288248 | 1.362509109626207 | 9.243711529314854 |
| H | 1.453594490219180 | 0.412845391918908 | 8.273141780828047 |
| C | 1.563258363143170 | 2.536163419618227 | 7.853025368834378 |
| C | 2.003320327019684 | 1.345685532130425 | 8.419470296783896 |
| O | 1.824222021398279 | 3.295015841788226 | 10.872466006337422 |
| O | 1.773520606305254 | 5.800084936926674 | 11.519313070651403 |
| O | 3.965811927617581 | 1.833717368874789 | 11.368416558417172 |
| O | 3.859271506244265 | -0.715137748434560 | 10.073170329759510 |
| O | 6.252099214331981 | -1.905251750287239 | 10.132357527915156 |
| O | 6.217096501930821 | 3.042617695420527 | 10.179637623058429 |
| O | 2.015812502958558 | 3.710037738334894 | 8.398168984135442 |
| Cu | 0.494080000000012 | 0.256759999999974 | 0.000000000000000 |
| Cu | 0.494080000000012 | 2.824179999999977 | 0.000000000000000 |
| Cu | 0.494080000000012 | 5.391680000000009 | 0.000000000000000 |
| Cu | 0.509993358491327 | 5.388478787464568 | 6.164980839007283 |

| | | | |
|---|---|---|---|
| Cu | 0.484482956080766 | 0.225518324225891 | 6.098700048246396 |
| Cu | 0.438696254711725 | 2.800352498832588 | 6.263035994956667 |
| Cu | 1.235229999999994 | 1.540470000000030 | 2.096320000000069 |
| Cu | 1.235229999999994 | 4.107970000000008 | 2.096320000000069 |
| Cu | 1.235229999999994 | 6.675390000000010 | 2.096320000000069 |
| Cu | 1.980189060828121 | 2.821087303708513 | 4.178083715114263 |
| Cu | 1.979013593086728 | 0.273874763003498 | 4.127431064797975 |
| Cu | 1.968160720612892 | 5.378993889161845 | 4.131664143082215 |
| Cu | 2.717599999999978 | -1.026989999999960 | 0.000000000000000 |
| Cu | 2.717599999999978 | 1.540429999999988 | 0.000000000000000 |
| Cu | 2.717599999999978 | 4.107930000000021 | 0.000000000000000 |
| Cu | 2.696474118438659 | 1.488642605006170 | 6.290732366031226 |
| Cu | 2.715345347496378 | -1.021926851516872 | 6.152316216029058 |
| Cu | 2.698699884339717 | 4.197911238776540 | 6.222930502430994 |
| Cu | 3.458750000000008 | 0.256720000000014 | 2.096320000000069 |
| Cu | 3.458750000000008 | 2.824219999999992 | 2.096320000000069 |
| Cu | 3.458750000000008 | 5.391639999999994 | 2.096320000000069 |
| Cu | 4.200150673979616 | -1.025369854962768 | 4.124611352148820 |
| Cu | 4.183497771671036 | 4.096503234987013 | 4.163585796154767 |
| Cu | 4.170332413421361 | 1.544200613979110 | 4.177654525015063 |
| Cu | 4.941050000000019 | -2.310700000000017 | 0.000000000000000 |
| Cu | 4.941050000000019 | 0.256719999999986 | 0.000000000000000 |
| Cu | 4.941050000000019 | 2.824219999999964 | 0.000000000000000 |
| Cu | 4.926293578111193 | -2.265415791895328 | 6.190322819073894 |
| Cu | 4.930877964085041 | 0.207441472959302 | 6.193515763536595 |
| Cu | 5.682200000000001 | -1.026990000000015 | 2.096320000000069 |
| Cu | 5.682200000000001 | 1.540510000000018 | 2.096320000000069 |
| Cu | 5.682200000000001 | 4.107930000000020 | 2.096320000000069 |
| Cu | 6.422782039605122 | 0.237691273840512 | 4.151613865376119 |
| Cu | 6.409261700586586 | 2.811736591148884 | 4.102961763648147 |
| Cu | 6.414187164187291 | -2.304818621627735 | 4.129058154553603 |
| Cd | 4.950711837894213 | 2.825891969305261 | 6.667351364917557 |

Cu111-Cd_CHCOH
60

| | | | |
|---|---|---|---|
| H | 1.429277352934968 | 4.670795160665285 | 8.421885904022236 |
| H | 0.998684515699341 | 5.342054996094346 | 9.850979417024414 |
| H | 2.637982495765853 | 3.874714606883862 | 10.080608341996530 |
| H | 0.603392032441453 | 6.581467015833649 | 11.671135914148778 |
| H | 3.580060139826125 | 2.433373803354682 | 10.601842434966597 |
| H | 3.078252431822120 | 0.633325943480919 | 10.088301391225826 |
| H | 4.290742671319952 | 3.991950229279813 | 10.153782621902620 |
| H | 4.581627056746644 | 0.763685593797093 | 10.409596639495035 |
| H | 5.359939116571911 | -0.371260144947931 | 8.461598885264324 |
| H | 5.672323785336014 | 4.417863725487306 | 8.620046949437063 |
| H | 5.616956928982617 | -2.080599194920014 | 9.630637234009431 |
| H | 6.529438094088644 | -0.111362935267202 | 9.456716433862377 |
| H | 6.552364798460158 | 3.354876441840246 | 10.424277444710315 |
| H | 2.882911565200939 | -1.156564812430425 | 8.762462388754445 |

| | | | |
|---|---|---|---|
| H | 1.189144442651949 | 1.929551260582151 | 8.681644101124601 |
| C | 1.381758602281798 | 1.443934831298038 | 7.717959428029631 |
| C | 2.022208792362012 | 0.223675149486437 | 7.713065871539805 |
| O | 2.363230408459930 | -0.348580456489850 | 8.963168053366804 |
| O | 1.354728947674833 | 4.505910331731659 | 9.403764942806010 |
| O | 0.141316876457733 | 6.346755456133067 | 10.848521400223278 |
| O | 3.465255082542903 | 3.550819661507196 | 10.579149528245955 |
| O | 3.703457281414887 | 1.121598639896025 | 10.702885824812206 |
| O | 5.586183897559420 | -0.454509458375017 | 9.430751953581240 |
| O | 5.586881688963593 | -3.094007992611365 | 9.591291707550948 |
| Cu | 0.494078305679993 | 0.256757570569980 | 0.000000000000000 |
| Cu | 0.494078305679993 | 2.824179229430008 | 0.000000000000000 |
| Cu | 0.494078305679993 | 5.391677911710007 | 0.000000000000000 |
| Cu | 0.527678034865612 | 5.294305770401903 | 6.174685757918417 |
| Cu | 0.443758894487131 | 0.218158389145610 | 6.259740184431526 |
| Cu | 0.539281582616165 | 2.749767362286106 | 6.312920744027442 |
| Cu | 1.235229116320017 | 1.540468399999967 | 2.096321626389977 |
| Cu | 1.235229116320017 | 4.107967082279965 | 2.096321626389977 |
| Cu | 1.235229116320017 | 6.675388741139993 | 2.096321626389977 |
| Cu | 1.991341937951959 | 2.813410744106035 | 4.144089691685854 |
| Cu | 1.969806359903712 | 0.252806046347613 | 4.128008808545477 |
| Cu | 1.983070355159505 | 5.394965813248237 | 4.137609741593113 |
| Cu | 2.717597441839991 | -1.026991770570019 | 0.000000000000000 |
| Cu | 2.717597441839991 | 1.540429888290010 | 0.000000000000000 |
| Cu | 2.717597441839991 | 4.107928570570007 | 0.000000000000000 |
| Cu | 2.781099755045214 | 1.552404165581390 | 6.192135272006845 |
| Cu | 2.689449860496038 | -1.003597943740725 | 6.238230840588155 |
| Cu | 2.712469026548518 | 4.155924047268765 | 6.184037347462073 |
| Cu | 3.458748252480015 | 0.256719058859968 | 2.096321626389977 |
| Cu | 3.458748252480015 | 2.824217741139966 | 2.096321626389977 |
| Cu | 3.458748252480015 | 5.391639399999995 | 2.096321626389977 |
| Cu | 4.187502651616144 | -1.039289455047924 | 4.137540853054849 |
| Cu | 4.206413127344970 | 4.094202000278689 | 4.136659497790650 |
| Cu | 4.198144685878843 | 1.531904312305213 | 4.145630584091105 |
| Cu | 4.941049873760016 | -2.310702600000033 | 0.000000000000000 |
| Cu | 4.941049873760016 | 0.256719058859995 | 0.000000000000000 |
| Cu | 4.941049873760016 | 2.824217741139993 | 0.000000000000000 |
| Cu | 4.897604323002375 | -2.288070520507491 | 6.201581135805969 |
| Cu | 5.005006139068587 | 0.166545533850300 | 6.156812949691463 |
| Cu | 5.682200684399993 | -1.026991770570018 | 2.096321626389977 |
| Cu | 5.682200684399993 | 1.540506911709979 | 2.096321626389977 |
| Cu | 5.682200684399993 | 4.107928570570007 | 2.096321626389977 |
| Cu | 6.447815146472494 | 0.216379665428928 | 4.137950517862300 |
| Cu | 6.434519860917309 | 2.827389430174723 | 4.158264601266690 |
| Cu | 6.424493782490447 | -2.331862891761940 | 4.157489211861635 |
| Cd | 4.954482059542771 | 2.773904931764020 | 6.668496070467524 |

Cu111-Cd_OCCH
59

| | | | |
|---|---|---|---|
| H | 1.947739785249141 | 2.306564943377050 | 9.912137030863695 |
| H | 2.567545766620360 | 3.487963422083606 | 10.906819496974093 |
| H | 3.403971298480998 | 1.852359672168951 | 10.685497463378987 |
| H | 3.051812986165543 | 5.359783120383203 | 10.397629689805209 |
| H | 4.371511478678694 | 0.047316438804215 | 10.145310319358037 |
| H | 4.179312281634880 | -1.622826730537358 | 8.479282936539482 |
| H | 5.367690884016511 | 1.282770455160313 | 10.203801206239538 |
| H | 4.982808564151741 | -2.165060888453125 | 9.736809679892984 |
| H | 0.195173122674124 | 1.286076482721477 | 9.740201687572624 |
| H | 0.285899649350885 | 5.696279051771855 | 8.704733033488896 |
| H | -0.005666859144625 | 6.787198157010295 | 9.834491311325747 |
| H | 6.487145618661886 | -3.015557778040487 | 11.233956801857856 |
| H | 1.514358802931607 | 5.158033936287140 | 10.589040916993868 |
| H | 2.263133013553550 | -0.162472893744172 | 8.636096640853768 |
| C | 1.312430304110640 | 1.534197434131558 | 7.752988383560854 |
| C | 1.977135378818492 | 0.315228095237237 | 7.690745044342886 |
| O | 2.513548198992079 | 2.421677270122620 | 10.772912321281126 |
| O | 2.389913659886612 | 4.892417086642303 | 10.980867249090970 |
| O | 4.512796126394830 | 0.930816873549277 | 10.575681340429590 |
| O | 4.229008193651078 | -1.568641631298658 | 9.465409966964627 |
| O | 6.254269801999206 | -3.183098800627813 | 10.302596918361941 |
| O | 0.161188250275558 | 5.810593275391062 | 9.674385061659422 |
| O | 0.938659650793323 | 2.216286772166699 | 8.814130680288759 |
| Cu | 0.494078305679993 | 0.256757570569980 | 0.000000000000000 |
| Cu | 0.494078305679993 | 2.824179229430008 | 0.000000000000000 |
| Cu | 0.494078305679993 | 5.391677911710007 | 0.000000000000000 |
| Cu | 0.519860058452866 | 5.334170304934446 | 6.153856379563762 |
| Cu | 0.470513606691603 | 0.220595832236644 | 6.241145050619394 |
| Cu | 0.534039930193976 | 2.779800234548017 | 6.259085642223745 |
| Cu | 1.235229116320017 | 1.540468399999967 | 2.096321626389977 |
| Cu | 1.235229116320017 | 4.107967082279965 | 2.096321626389977 |
| Cu | 1.235229116320017 | 6.675388741139993 | 2.096321626389977 |
| Cu | 2.000019960602311 | 2.818259117969760 | 4.150269709361166 |
| Cu | 1.978820815967810 | 0.260272799783769 | 4.128914396071697 |
| Cu | 1.986355756127375 | 5.406316124169554 | 4.138194331055695 |
| Cu | 2.717597441839991 | -1.026991770570019 | 0.000000000000000 |
| Cu | 2.717597441839991 | 1.540429888290010 | 0.000000000000000 |
| Cu | 2.717597441839991 | 4.107928570570007 | 0.000000000000000 |
| Cu | 2.783189585682091 | 1.561479121827209 | 6.214131991485032 |
| Cu | 2.704029664102554 | -0.985301062226702 | 6.268459574677706 |
| Cu | 2.711413477497496 | 4.175107332181172 | 6.177978668604646 |
| Cu | 3.458748252480015 | 0.256719058859968 | 2.096321626389977 |
| Cu | 3.458748252480015 | 2.824217741139966 | 2.096321626389977 |
| Cu | 3.458748252480015 | 5.391639399999995 | 2.096321626389977 |
| Cu | 4.190679271216035 | -1.021198287543093 | 4.138689594303079 |
| Cu | 4.207374573082529 | 4.098732194849060 | 4.133568305245547 |
| Cu | 4.199908187435676 | 1.542635748748332 | 4.147104401593473 |
| Cu | 4.941049873760016 | -2.310702600000033 | 0.000000000000000 |
| Cu | 4.941049873760016 | 0.256719058859995 | 0.000000000000000 |
| Cu | 4.941049873760016 | 2.824217741139993 | 0.000000000000000 |

| | | | |
|---|---|---|---|
| Cu | 4.916070235397337 | -2.271549836006251 | 6.154930084045211 |
| Cu | 4.994544726026335 | 0.202887182574347 | 6.177987667720884 |
| Cu | 5.682200684399993 | -1.026991770570018 | 2.096321626389977 |
| Cu | 5.682200684399993 | 1.540506911709979 | 2.096321626389977 |
| Cu | 5.682200684399993 | 4.107928570570007 | 2.096321626389977 |
| Cu | 6.441430552785567 | 0.227599690666394 | 4.138561036357856 |
| Cu | 6.430207251827980 | 2.838202921038554 | 4.146170681419941 |
| Cu | 6.433006765622928 | -2.332070839696204 | 4.149520016255824 |
| Cd | 4.949916808154451 | 2.826520798722587 | 6.742283133672310 |

Cu111-Cd_OCCOH
60

| | | | |
|---|---|---|---|
| H | 1.705420274644371 | 3.570553359623781 | 10.427368978934082 |
| H | 1.888355134705997 | 4.778246036855230 | 11.622652765647940 |
| H | 3.012874656776637 | 3.234159574235695 | 11.564326939487525 |
| H | 2.419836626083524 | -0.922261081280218 | 11.756027152009572 |
| H | 3.891989079762595 | 1.605149063973623 | 11.416089403191922 |
| H | 3.067301689798334 | 0.148980849410527 | 10.026189663515234 |
| H | 4.860163706288289 | 2.915224048170719 | 11.269557524222067 |
| H | 4.389005636251910 | -0.438236775226865 | 10.789000862863837 |
| H | 6.441172198737716 | -1.160282831245587 | 10.195132096219748 |
| H | 0.156444226258648 | 7.548520779306712 | 9.986500371042078 |
| H | 6.081040643509887 | -3.051652523729523 | 10.981715045218133 |
| H | 6.337501334957315 | -0.978502024848722 | 11.746817783479083 |
| H | 0.860347676896053 | 6.630271691974025 | 11.779392407125508 |
| H | 1.763737767074985 | -0.781090482670899 | 8.465928586350675 |
| C | 1.047462976735337 | 2.319838993384312 | 8.063985908284268 |
| C | 1.415955620457038 | 1.001494176844701 | 7.802365316921743 |
| O | 1.649532737260295 | 0.092222638486386 | 8.910091296136908 |
| O | 1.985872919467260 | 3.763103803789590 | 11.376156312148597 |
| O | 1.692580112520398 | 6.177976066786377 | 12.070098005252843 |
| O | 4.051207102664519 | 2.551508711539472 | 11.736768937381854 |
| O | 3.556783866404728 | 0.109660158645351 | 10.888497352669196 |
| O | 5.826672452923584 | -1.333152668477773 | 10.992892697719927 |
| O | 6.267706601960062 | 3.685376428951218 | 10.803759162422024 |
| O | 0.913574216946419 | 2.974925681945282 | 9.158847673446658 |
| Cu | 0.494078305679993 | 0.256757570569980 | 0.000000000000000 |
| Cu | 0.494078305679993 | 2.824179229430008 | 0.000000000000000 |
| Cu | 0.494078305679993 | 5.391677911710007 | 0.000000000000000 |
| Cu | 0.527170778317564 | 5.380892965171053 | 6.173540107453646 |
| Cu | 0.521191072140819 | 0.238016033505645 | 6.201911292569622 |
| Cu | 0.453837522976698 | 2.901265256326763 | 6.261003100941251 |
| Cu | 1.235229116320017 | 1.540468399999967 | 2.096321626389977 |
| Cu | 1.235229116320017 | 4.107967082279965 | 2.096321626389977 |
| Cu | 1.235229116320017 | 6.675388741139993 | 2.096321626389977 |
| Cu | 1.975230274092506 | 2.807220214127550 | 4.169158907690198 |
| Cu | 1.972720709190952 | 0.263377239960684 | 4.120181780810102 |
| Cu | 1.964610729424160 | 5.379072500700401 | 4.122370861624721 |
| Cu | 2.717597441839991 | -1.026991770570019 | 0.000000000000000 |

| | | | |
|---|---|---|---|
| Cu | 2.717597441839991 | 1.540429888290010 | 0.000000000000000 |
| Cu | 2.717597441839991 | 4.107928570570007 | 0.000000000000000 |
| Cu | 2.708038668679032 | 1.450686320270853 | 6.285857544632772 |
| Cu | 2.739212516846076 | -1.082747140907836 | 6.131257485470250 |
| Cu | 2.670323957454381 | 4.122502497360761 | 6.215164778095303 |
| Cu | 3.458748252480015 | 0.256719058859968 | 2.096321626389977 |
| Cu | 3.458748252480015 | 2.824217741139966 | 2.096321626389977 |
| Cu | 3.458748252480015 | 5.391639399999995 | 2.096321626389977 |
| Cu | 4.206672771037345 | -1.023549063068675 | 4.119228390246183 |
| Cu | 4.192781131458017 | 4.098924932420804 | 4.160329062990634 |
| Cu | 4.169291731225388 | 1.546947004575501 | 4.171086662411223 |
| Cu | 4.941049873760016 | -2.310702600000033 | 0.000000000000000 |
| Cu | 4.941049873760016 | 0.256719058859995 | 0.000000000000000 |
| Cu | 4.941049873760016 | 2.824217741139993 | 0.000000000000000 |
| Cu | 4.921722282964734 | -2.277126604389484 | 6.186906727456221 |
| Cu | 4.931613257484038 | 0.207398253077674 | 6.172793265349101 |
| Cu | 5.682200684399993 | -1.026991770570018 | 2.096321626389977 |
| Cu | 5.682200684399993 | 1.540506911709979 | 2.096321626389977 |
| Cu | 5.682200684399993 | 4.107928570570007 | 2.096321626389977 |
| Cu | 6.421247923718389 | 0.251113978682525 | 4.129226197869794 |
| Cu | 6.419438790415728 | 2.842110132645361 | 4.151921881993440 |
| Cu | 6.422374129088271 | -2.309230490377655 | 4.152125130397178 |
| Cd | 4.931814227234265 | 2.813169352673789 | 6.696931855961247 |

Cu111-Au_H
56

| | | | |
|---|---|---|---|
| H | 1.376247271267131 | 4.188244264285871 | 8.368592781669632 |
| H | 1.218321482345698 | 5.516278705891679 | 9.868488900565575 |
| H | 2.098337745609264 | 3.603996371669709 | 9.706220044053849 |
| H | 2.029987952762445 | -0.684110364037154 | 9.940136454442195 |
| H | 3.281598708460732 | 1.885291734360961 | 10.014521810948331 |
| H | 3.109675144651017 | 0.257369274746449 | 8.477149574540629 |
| H | 4.147064856101136 | 3.210285491057070 | 10.008852191734167 |
| H | 4.121984455636389 | -0.320063358384537 | 9.528047444754680 |
| H | 5.570716388643901 | -1.164401458272007 | 8.297853823694140 |
| H | 5.680132032497393 | 3.698450740563033 | 8.469748859844270 |
| H | 5.754563320545802 | -2.828016299976875 | 9.519835746439245 |
| H | 6.458083245726929 | -0.610426227030947 | 9.456275756955215 |
| H | 0.338917945332723 | 7.006299001912342 | 9.931573902557355 |
| H | 4.117590284410925 | 1.409389699081387 | 7.193945733845608 |
| O | 1.271904117631807 | 4.142882295003186 | 9.358485014594740 |
| O | 1.188187530150741 | 6.501645880079171 | 10.254003013573646 |
| O | 3.265935184560250 | 2.840420107069362 | 10.269177554865776 |
| O | 3.226130607124574 | 0.133780914993625 | 9.456367544222751 |
| O | 5.614747579322951 | -1.131229233085583 | 9.297481675704999 |
| O | 5.748959401966798 | 3.870568478321013 | 9.455498652649158 |
| Cu | 0.494078305679993 | 0.256757570569980 | 0.000000000000000 |
| Cu | 0.494078305679993 | 2.824179229430008 | 0.000000000000000 |
| Cu | 0.494078305679993 | 5.391677911710007 | 0.000000000000000 |

| | | | |
|---|---|---|---|
| Cu | 0.548255043727712 | 5.378207381792689 | 6.201356440059862 |
| Cu | 0.540633342316041 | 0.290238286843435 | 6.211726473617299 |
| Cu | 0.497504784964446 | 2.834873919262903 | 6.185692781683333 |
| Cu | 1.235229116320017 | 1.540468399999967 | 2.096321626389977 |
| Cu | 1.235229116320017 | 4.107967082279965 | 2.096321626389977 |
| Cu | 1.235229116320017 | 6.675388741139993 | 2.096321626389977 |
| Cu | 1.993838998447839 | 2.818520073696232 | 4.156793959042474 |
| Cu | 1.982397261018426 | 0.266389181920271 | 4.148129677680631 |
| Cu | 1.977421761961299 | 5.395843335213030 | 4.129501151523876 |
| Cu | 2.717597441839991 | -1.026991770570019 | 0.000000000000000 |
| Cu | 2.717597441839991 | 1.540429888290010 | 0.000000000000000 |
| Cu | 2.717597441839991 | 4.107928570570007 | 0.000000000000000 |
| Cu | 2.664898994314031 | 1.519659319444718 | 6.240605354297815 |
| Cu | 2.711170622059227 | -1.038579436103084 | 6.200061803530026 |
| Cu | 2.679473017003907 | 4.148418310687100 | 6.196434244147659 |
| Cu | 3.458748252480015 | 0.256719058859968 | 2.096321626389977 |
| Cu | 3.458748252480015 | 2.824217741139966 | 2.096321626389977 |
| Cu | 3.458748252480015 | 5.391639399999995 | 2.096321626389977 |
| Cu | 4.201962383289544 | -1.014654680649552 | 4.139369364600336 |
| Cu | 4.194155139932035 | 4.117679759780608 | 4.124056306433252 |
| Cu | 4.195391338059346 | 1.533705949760410 | 4.105342328324931 |
| Cu | 4.941049873760016 | -2.310702600000033 | 0.000000000000000 |
| Cu | 4.941049873760016 | 0.256719058859995 | 0.000000000000000 |
| Cu | 4.941049873760016 | 2.824217741139993 | 0.000000000000000 |
| Cu | 4.943716982239186 | -2.259903773356437 | 6.183772470449326 |
| Cu | 4.940098751956681 | 0.201852863479987 | 6.232303026069745 |
| Cu | 5.682200684399993 | -1.026991770570018 | 2.096321626389977 |
| Cu | 5.682200684399993 | 1.540506911709979 | 2.096321626389977 |
| Cu | 5.682200684399993 | 4.107928570570007 | 2.096321626389977 |
| Cu | 6.409441826997728 | 0.271277780230297 | 4.155029960657006 |
| Cu | 6.435970596312333 | 2.826414432309010 | 4.129605600935585 |
| Cu | 6.426646003218461 | -2.311698106370106 | 4.136342349384524 |
| Au | 4.966798872382127 | 2.866081188462676 | 6.392400715983375 |

Cu111-Co_H
56

| | | | |
|---|---|---|---|
| H | 1.364842499796352 | 4.269673197013597 | 8.322107815851480 |
| H | 1.241486637901491 | 5.610787626832606 | 9.776778168503252 |
| H | 2.114427652062024 | 3.685679950410806 | 9.648892786480815 |
| H | 2.039589837961380 | -0.574324803644685 | 9.788586114755184 |
| H | 3.283858978491092 | 1.966576035076802 | 9.905973049634285 |
| H | 3.170021163665985 | 0.356230760842243 | 8.329836708791165 |
| H | 4.154102517164613 | 3.282987927430428 | 9.876865362725500 |
| H | 4.144691784633638 | -0.218787313120034 | 9.421832867345183 |
| H | 5.588144202432652 | -1.071220153509617 | 8.232381837062837 |
| H | 5.666980246834610 | 3.756360855204817 | 8.287740653435579 |
| H | 5.744395849911869 | -2.789509815753537 | 9.364195431978571 |
| H | 6.465519374891882 | -0.550728228823172 | 9.412710218542141 |
| H | 0.345847740224789 | 7.088063009162726 | 9.764806232053271 |

| | | | |
|---|---|---|---|
| H | 3.643662104017710 | 2.836866895019683 | 7.069971761765264 |
| O | 1.279335235347158 | 4.225618231450277 | 9.313597523358611 |
| O | 1.202918353459128 | 6.615240580182403 | 10.114162219398516 |
| O | 3.282846071415501 | 2.918297121969797 | 10.177964578813134 |
| O | 3.258495889568253 | 0.246448013094073 | 9.317204860221951 |
| O | 5.626059318244856 | -1.069461327714892 | 9.232513250242420 |
| O | 5.735263721844358 | 3.911481895024662 | 9.276980575536676 |
| Co | 4.948076396328370 | 2.829421482788456 | 6.124893538429830 |
| Cu | 0.494078305679993 | 0.256757570569980 | 0.000000000000000 |
| Cu | 0.494078305679993 | 2.824179229430008 | 0.000000000000000 |
| Cu | 0.494078305679993 | 5.391677911710007 | 0.000000000000000 |
| Cu | 0.475819284424311 | 5.414264567882284 | 6.186511744173072 |
| Cu | 0.483591567901358 | 0.249586812497005 | 6.191723950069362 |
| Cu | 0.481071593493556 | 2.832661256579381 | 6.172999807150853 |
| Cu | 1.235229116320017 | 1.540468399999967 | 2.096321626389977 |
| Cu | 1.235229116320017 | 4.107967082279965 | 2.096321626389977 |
| Cu | 1.235229116320017 | 6.675388741139993 | 2.096321626389977 |
| Cu | 1.972996947750060 | 2.827400482909671 | 4.135591547353861 |
| Cu | 1.983003823960029 | 0.270649182851025 | 4.152246244657420 |
| Cu | 1.983957569483296 | 5.385421143068819 | 4.149144651919625 |
| Cu | 2.717597441839991 | -1.026991770570019 | 0.000000000000000 |
| Cu | 2.717597441839991 | 1.540429888290010 | 0.000000000000000 |
| Cu | 2.717597441839991 | 4.107928570570007 | 0.000000000000000 |
| Cu | 2.730438135633022 | 1.547456460879081 | 6.210571767596950 |
| Cu | 2.717891918105605 | -1.024273951743239 | 6.197472255289570 |
| Cu | 2.732214460328547 | 4.118483049940587 | 6.211692768536285 |
| Cu | 3.458748252480015 | 0.256719058859968 | 2.096321626389977 |
| Cu | 3.458748252480015 | 2.824217741139966 | 2.096321626389977 |
| Cu | 3.458748252480015 | 5.391639399999995 | 2.096321626389977 |
| Cu | 4.200585856562546 | -1.024315380436072 | 4.140219043023689 |
| Cu | 4.206255131499780 | 4.102603770157233 | 4.127845570732107 |
| Cu | 4.206258520216928 | 1.550134717097302 | 4.126370792469216 |
| Cu | 4.941049873760016 | -2.310702600000033 | 0.000000000000000 |
| Cu | 4.941049873760016 | 0.256719058859995 | 0.000000000000000 |
| Cu | 4.941049873760016 | 2.824217741139993 | 0.000000000000000 |
| Cu | 4.946659902671912 | -2.326523056472020 | 6.173863738734494 |
| Cu | 4.943673509434311 | 0.282070066418726 | 6.181389776487170 |
| Cu | 5.682200684399993 | -1.026991770570018 | 2.096321626389977 |
| Cu | 5.682200684399993 | 1.540506911709979 | 2.096321626389977 |
| Cu | 5.682200684399993 | 4.107928570570007 | 2.096321626389977 |
| Cu | 6.423570233125773 | 0.264647644277779 | 4.134018407510109 |
| Cu | 6.422360205876565 | 2.829215277240608 | 4.130939297130162 |
| Cu | 6.427589606422811 | -2.309649119433812 | 4.134450630527089 |

Cu111-Ni_H
56

| | | | |
|---|---|---|---|
| H | 1.337222824781366 | 4.211059096975008 | 8.320743766373234 |
| H | 1.210399321464082 | 5.554597576500533 | 9.771631200551635 |
| H | 2.085645375349479 | 3.631502462452485 | 9.647979273983969 |

| | | | |
|---|---|---|---|
| H | 2.007637129308411 | -0.629204514998368 | 9.781554005588674 |
| H | 3.256669485223524 | 1.913379079552291 | 9.896127124429471 |
| H | 3.125314482179321 | 0.301713729960662 | 8.318201904980826 |
| H | 4.126752056289731 | 3.230205397773021 | 9.870793705297151 |
| H | 4.108730461029540 | -0.271799177941192 | 9.405143099750619 |
| H | 5.565944150694491 | -1.118161752848247 | 8.219111195418451 |
| H | 5.643146860777298 | 3.697514163896526 | 8.277328770649213 |
| H | 5.715890641313870 | -2.840457103965945 | 9.347176890125539 |
| H | 6.433628037382710 | -0.605465149941031 | 9.409020183579152 |
| H | 0.315853629841900 | 7.033817690372113 | 9.758231430995695 |
| H | 3.611308049655714 | 2.829159181862503 | 7.049787226327440 |
| O | 1.250355688268894 | 4.170660303779401 | 9.312318456273943 |
| O | 1.171614409768970 | 6.559969723107811 | 10.108544237204447 |
| O | 3.256721052285439 | 2.863944278621104 | 10.172746995555501 |
| O | 3.222225105413000 | 0.193949842239876 | 9.305553408138900 |
| O | 5.594112931252760 | -1.120975385492445 | 9.220432090980376 |
| O | 5.707626225955401 | 3.859867186267289 | 9.265199227293690 |
| Ni | 4.955378098335868 | 2.829989265267853 | 6.147224673358456 |
| Cu | 0.494078305679993 | 0.256757570569980 | 0.000000000000000 |
| Cu | 0.494078305679993 | 2.824179229430008 | 0.000000000000000 |
| Cu | 0.494078305679993 | 5.391677911710007 | 0.000000000000000 |
| Cu | 0.485352865915626 | 5.408100116513461 | 6.186378544745947 |
| Cu | 0.494940293354908 | 0.255565833410809 | 6.188363431876216 |
| Cu | 0.485140218272123 | 2.831208044344335 | 6.170441859647505 |
| Cu | 1.235229116320017 | 1.540468399999967 | 2.096321626389977 |
| Cu | 1.235229116320017 | 4.107967082279965 | 2.096321626389977 |
| Cu | 1.235229116320017 | 6.675388741139993 | 2.096321626389977 |
| Cu | 1.975429357758593 | 2.826951372329774 | 4.131965917826528 |
| Cu | 1.985703824788313 | 0.270152647702539 | 4.148609763786967 |
| Cu | 1.986322302706767 | 5.383780328885277 | 4.148085152977139 |
| Cu | 2.717597441839991 | -1.026991770570019 | 0.000000000000000 |
| Cu | 2.717597441839991 | 1.540429888290010 | 0.000000000000000 |
| Cu | 2.717597441839991 | 4.107928570570007 | 0.000000000000000 |
| Cu | 2.727097300983469 | 1.543968929770465 | 6.207520499006928 |
| Cu | 2.721063598467455 | -1.025624646523823 | 6.191014767030565 |
| Cu | 2.729621644278373 | 4.119519327437684 | 6.211346850326273 |
| Cu | 3.458748252480015 | 0.256719058859968 | 2.096321626389977 |
| Cu | 3.458748252480015 | 2.824217741139966 | 2.096321626389977 |
| Cu | 3.458748252480015 | 5.391639399999995 | 2.096321626389977 |
| Cu | 4.201402426716645 | -1.024281821194997 | 4.138854632983069 |
| Cu | 4.204589969407139 | 4.106781904228688 | 4.124792652058452 |
| Cu | 4.206172779947052 | 1.545185320898927 | 4.125139617406081 |
| Cu | 4.941049873760016 | -2.310702600000033 | 0.000000000000000 |
| Cu | 4.941049873760016 | 0.256719058859995 | 0.000000000000000 |
| Cu | 4.941049873760016 | 2.824217741139993 | 0.000000000000000 |
| Cu | 4.949830442600031 | -2.318539440513902 | 6.165300412161581 |
| Cu | 4.947379099765492 | 0.272854484924357 | 6.178617465418363 |
| Cu | 5.682200684399993 | -1.026991770570018 | 2.096321626389977 |
| Cu | 5.682200684399993 | 1.540506911709979 | 2.096321626389977 |
| Cu | 5.682200684399993 | 4.107928570570007 | 2.096321626389977 |

| | | | |
|---|---|---|---|
| Cu | 6.424485779457004 | 0.265198261801848 | 4.134815320564039 |
| Cu | 6.417712666057034 | 2.827760206664367 | 4.138290637111511 |
| Cu | 6.429046932238755 | -2.311708535633331 | 4.133539258620566 |

Cu111-Pd_H
56

| | | | |
|---|---|---|---|
| H | 1.393866831225608 | 4.269068956492929 | 8.345400804258404 |
| H | 1.256959348183034 | 5.625445529330220 | 9.792318048570589 |
| H | 2.122990491734085 | 3.694451653849925 | 9.684373522233987 |
| H | 2.057543709163177 | -0.560541811219056 | 9.798924942130071 |
| H | 3.296799197507149 | 1.978219460633696 | 9.936494608689159 |
| H | 3.171603776089767 | 0.374705619962561 | 8.337108310551191 |
| H | 4.160211663434179 | 3.297991033549886 | 9.949752524078699 |
| H | 4.156459792784627 | -0.203163837365969 | 9.419571452968935 |
| H | 5.611493365438747 | -1.063031255118532 | 8.236223153040116 |
| H | 5.654413625858981 | 3.745165629395457 | 8.350037378181327 |
| H | 5.758487960995257 | -2.768891675397708 | 9.395708385476018 |
| H | 6.478317899461932 | -0.534581006645999 | 9.421350645764605 |
| H | 0.361740613784722 | 7.105890149248178 | 9.798865574398468 |
| H | 3.426046145970821 | 2.830965348601460 | 7.129380641504783 |
| O | 1.294785152267389 | 4.234664073053500 | 9.335523101138868 |
| O | 1.223564824325595 | 6.628148171073021 | 10.131742412171302 |
| O | 3.287039217117492 | 2.923221190702795 | 10.230596124890923 |
| O | 3.269288632230326 | 0.262167358941622 | 9.323025585451905 |
| O | 5.639799207584832 | -1.053586789230442 | 9.236898663790646 |
| O | 5.747149739457042 | 3.930036630815654 | 9.334061286545390 |
| Cu | 0.494078305679993 | 0.256757570569980 | 0.000000000000000 |
| Cu | 0.494078305679993 | 2.824179229430008 | 0.000000000000000 |
| Cu | 0.494078305679993 | 5.391677911710007 | 0.000000000000000 |
| Cu | 0.530764703713257 | 5.385420730907996 | 6.185635334028993 |
| Cu | 0.537246013150343 | 0.276284702909611 | 6.191884621008353 |
| Cu | 0.481732301657114 | 2.831293079744170 | 6.172958661925689 |
| Cu | 1.235229116320017 | 1.540468399999967 | 2.096321626389977 |
| Cu | 1.235229116320017 | 4.107967082279965 | 2.096321626389977 |
| Cu | 1.235229116320017 | 6.675388741139993 | 2.096321626389977 |
| Cu | 1.978467946039613 | 2.827063802648346 | 4.134082660013076 |
| Cu | 1.986200022528259 | 0.270240371765558 | 4.140534011422014 |
| Cu | 1.988432514103859 | 5.383754019802652 | 4.139559408779484 |
| Cu | 2.717597441839991 | -1.026991770570019 | 0.000000000000000 |
| Cu | 2.717597441839991 | 1.540429888290010 | 0.000000000000000 |
| Cu | 2.717597441839991 | 4.107928570570007 | 0.000000000000000 |
| Cu | 2.681395201488717 | 1.524556450970463 | 6.212712981559287 |
| Cu | 2.720968133955076 | -1.023307358001522 | 6.190402591210616 |
| Cu | 2.686026184910844 | 4.141324522481686 | 6.217192575602535 |
| Cu | 3.458748252480015 | 0.256719058859968 | 2.096321626389977 |
| Cu | 3.458748252480015 | 2.824217741139966 | 2.096321626389977 |
| Cu | 3.458748252480015 | 5.391639399999995 | 2.096321626389977 |
| Cu | 4.199603796869485 | -1.025523672387744 | 4.132038788317701 |
| Cu | 4.198252688907712 | 4.117324839690345 | 4.118647288814430 |

| | | | |
|---|---|---|---|
| Cu | 4.197885466590899 | 1.533168237723977 | 4.116611797698516 |
| Cu | 4.941049873760016 | -2.310702600000033 | 0.000000000000000 |
| Cu | 4.941049873760016 | 0.256719058859995 | 0.000000000000000 |
| Cu | 4.941049873760016 | 2.824217741139993 | 0.000000000000000 |
| Cu | 4.943606638897179 | -2.272308619112947 | 6.176352806362408 |
| Cu | 4.941820718004146 | 0.225858046811911 | 6.181618565298994 |
| Cu | 5.682200684399993 | -1.026991770570018 | 2.096321626389977 |
| Cu | 5.682200684399993 | 1.540506911709979 | 2.096321626389977 |
| Cu | 5.682200684399993 | 4.107928570570007 | 2.096321626389977 |
| Cu | 6.420581318750111 | 0.270942844423408 | 4.141596616299253 |
| Cu | 6.432241624357574 | 2.827157672275145 | 4.127012490907505 |
| Cu | 6.424484940896022 | -2.318970719654979 | 4.143232842751331 |
| Pd | 4.969955330724286 | 2.828654687298510 | 6.284892908001031 |

Cu111-Pt_H
56

| | | | |
|---|---|---|---|
| H | 1.305100853903857 | 4.140551980931142 | 8.345334857142083 |
| H | 1.160065300293423 | 5.501054596005409 | 9.794187369629611 |
| H | 2.028883580070971 | 3.564723015160327 | 9.687660981617409 |
| H | 1.957057491902711 | -0.687820489328487 | 9.798887215673307 |
| H | 3.202837213683251 | 1.847700779585378 | 9.952221769577722 |
| H | 3.076444619799583 | 0.260992076258702 | 8.340461774446229 |
| H | 4.069076689559136 | 3.166367679269491 | 9.973657857393428 |
| H | 4.062583310915070 | -0.327660014541524 | 9.417038364135985 |
| H | 5.518153556985415 | -1.188299625951865 | 8.234970474964372 |
| H | 5.541285393864721 | 3.583512767803602 | 8.368217367076396 |
| H | 5.663052927212463 | -2.909942797906179 | 9.399046401094372 |
| H | 6.383543549763663 | -0.665010861303002 | 9.421712717185066 |
| H | 0.255684304046437 | 6.980570284550369 | 9.801877942925705 |
| H | 3.525676063427603 | 2.820569093097002 | 7.189956904721974 |
| O | 1.201999489533793 | 4.103114262123552 | 9.334766276376198 |
| O | 1.125185391575109 | 6.501540752933813 | 10.131417485317243 |
| O | 3.190593300023166 | 2.794063496659824 | 10.242091915598657 |
| O | 3.175982606028143 | 0.139336949935603 | 9.324339118417573 |
| O | 5.543979031995731 | -1.181798125179226 | 9.235618352267199 |
| O | 5.651661856209010 | 3.789826045770945 | 9.352706456480583 |
| Cu | 0.494078305679993 | 0.256757570569980 | 0.000000000000000 |
| Cu | 0.494078305679993 | 2.824179229430008 | 0.000000000000000 |
| Cu | 0.494078305679993 | 5.391677911710007 | 0.000000000000000 |
| Cu | 0.517545228441001 | 5.384967194239517 | 6.196654757126439 |
| Cu | 0.525242015697833 | 0.269220689627020 | 6.202179352272683 |
| Cu | 0.478159024593136 | 2.826346759715524 | 6.173826243960182 |
| Cu | 1.235229116320017 | 1.540468399999967 | 2.096321626389977 |
| Cu | 1.235229116320017 | 4.107967082279965 | 2.096321626389977 |
| Cu | 1.235229116320017 | 6.675388741139993 | 2.096321626389977 |
| Cu | 1.975559635106246 | 2.824929440549353 | 4.139172939589926 |
| Cu | 1.980818093108604 | 0.268144987976118 | 4.143954651596728 |
| Cu | 1.982833860477174 | 5.381554569713420 | 4.142412803167042 |
| Cu | 2.717597441839991 | -1.026991770570019 | 0.000000000000000 |

| | | | |
|---|---|---|---|
| Cu | 2.717597441839991 | 1.540429888290010 | 0.000000000000000 |
| Cu | 2.717597441839991 | 4.107928570570007 | 0.000000000000000 |
| Cu | 2.673602648977762 | 1.514995330070499 | 6.219062830777840 |
| Cu | 2.714848698844170 | -1.028245303548079 | 6.187264484386986 |
| Cu | 2.674810871411186 | 4.141042975372050 | 6.222798403314549 |
| Cu | 3.458748252480015 | 0.256719058859968 | 2.096321626389977 |
| Cu | 3.458748252480015 | 2.824217741139966 | 2.096321626389977 |
| Cu | 3.458748252480015 | 5.391639399999995 | 2.096321626389977 |
| Cu | 4.199315303851052 | -1.025499027187170 | 4.136674103506337 |
| Cu | 4.191410108753510 | 4.118945250699196 | 4.111565172921460 |
| Cu | 4.191689671305456 | 1.526664768540378 | 4.111311550840171 |
| Cu | 4.941049873760016 | -2.310702600000033 | 0.000000000000000 |
| Cu | 4.941049873760016 | 0.256719058859995 | 0.000000000000000 |
| Cu | 4.941049873760016 | 2.824217741139993 | 0.000000000000000 |
| Cu | 4.937577005465675 | -2.278087660989163 | 6.177097510388606 |
| Cu | 4.937464292936919 | 0.227419494393138 | 6.195401344311010 |
| Cu | 5.682200684399993 | -1.026991770570018 | 2.096321626389977 |
| Cu | 5.682200684399993 | 1.540506911709979 | 2.096321626389977 |
| Cu | 5.682200684399993 | 4.107928570570007 | 2.096321626389977 |
| Cu | 6.418594861179989 | 0.264303391415521 | 4.140142958218228 |
| Cu | 6.435608878517117 | 2.825056687421742 | 4.121806321693699 |
| Cu | 6.423551899973723 | -2.315832957563225 | 4.139008762396896 |
| Pt | 4.963329257654344 | 2.827885827211878 | 6.265070403636575 |

Cu111-Zn_H
56

| | | | |
|---|---|---|---|
| H | 1.710476997242488 | 4.830313959357290 | 8.405811773277584 |
| H | 1.569203306618458 | 6.116521792225182 | 9.902548265142840 |
| H | 2.430972494010769 | 4.197703636741513 | 9.717632255462407 |
| H | 2.380249615688462 | -0.080650826414498 | 9.923809176617224 |
| H | 3.605821123516028 | 2.453811376405317 | 9.946587118217179 |
| H | 3.459467700025512 | 0.763582840540398 | 8.408221819333544 |
| H | 4.478205713518453 | 3.773507543803020 | 9.962455686607129 |
| H | 4.473909117588372 | 0.252576108657306 | 9.495954936702351 |
| H | 5.917790680163010 | -0.596237754415887 | 8.293719386808730 |
| H | 6.070708941512176 | -3.403226488931440 | 8.420568634478068 |
| H | 6.087109810675742 | -2.258761545882217 | 9.496422456285831 |
| H | 0.130197704825727 | 3.816877158424222 | 9.457543637217757 |
| H | 0.691641712827765 | -0.102680677562809 | 9.925468620184434 |
| H | 3.098935749219067 | 2.824686088076912 | 7.258714423956111 |
| O | 1.606980623264025 | 4.755008349314753 | 9.393625209615623 |
| O | 1.541705594862772 | -0.586577055611828 | 10.264480321145211 |
| O | 3.602232506420482 | 3.400775325043337 | 10.234166949822198 |
| O | 3.581823722581831 | 0.707690788479324 | 9.396173299797367 |
| O | 5.966632870366468 | -0.567140993698499 | 9.294097415773361 |
| O | 6.083468340222344 | -3.262000264079428 | 9.408702208190546 |
| Cu | 0.494078305679993 | 0.256757570569980 | 0.000000000000000 |
| Cu | 0.494078305679993 | 2.824179229430008 | 0.000000000000000 |
| Cu | 0.494078305679993 | 5.391677911710007 | 0.000000000000000 |

| | | | |
|---|---|---|---|
| Cu | 0.491762756431807 | 5.392481673245580 | 6.206697046343386 |
| Cu | 0.496563308793031 | 0.256760690419812 | 6.210835926764890 |
| Cu | 0.477914726952684 | 2.826522526351696 | 6.185467232052912 |
| Cu | 1.235229116320017 | 1.540468399999967 | 2.096321626389977 |
| Cu | 1.235229116320017 | 4.107967082279965 | 2.096321626389977 |
| Cu | 1.235229116320017 | 6.675388741139993 | 2.096321626389977 |
| Cu | 1.981511346547913 | 2.825369576596374 | 4.131432260841605 |
| Cu | 1.984425045125116 | 0.269086147896259 | 4.165192151207638 |
| Cu | 1.985801385559093 | 5.382677940868587 | 4.164852165100679 |
| Cu | 2.717597441839991 | -1.026991770570019 | 0.000000000000000 |
| Cu | 2.717597441839991 | 1.540429888290010 | 0.000000000000000 |
| Cu | 2.717597441839991 | 4.107928570570007 | 0.000000000000000 |
| Cu | 2.700425555723117 | 1.538450693128973 | 6.240762541605084 |
| Cu | 2.720724431849189 | -1.024563760231617 | 6.226123421979621 |
| Cu | 2.705549310897049 | 4.120870463329468 | 6.244513207187891 |
| Cu | 3.458748252480015 | 0.256719058859968 | 2.096321626389977 |
| Cu | 3.458748252480015 | 2.824217741139966 | 2.096321626389977 |
| Cu | 3.458748252480015 | 5.391639399999995 | 2.096321626389977 |
| Cu | 4.199235235960376 | -1.030795043288094 | 4.138214930181793 |
| Cu | 4.202323186043894 | 4.108153217168844 | 4.150620879978772 |
| Cu | 4.202590902230118 | 1.546869195040404 | 4.143193031249186 |
| Cu | 4.941049873760016 | -2.310702600000033 | 0.000000000000000 |
| Cu | 4.941049873760016 | 0.256719058859995 | 0.000000000000000 |
| Cu | 4.941049873760016 | 2.824217741139993 | 0.000000000000000 |
| Cu | 4.952535247038629 | -2.288379573406592 | 6.209286592056749 |
| Cu | 4.944700307184819 | 0.241970073070958 | 6.179142407787186 |
| Cu | 5.682200684399993 | -1.026991770570018 | 2.096321626389977 |
| Cu | 5.682200684399993 | 1.540506911709979 | 2.096321626389977 |
| Cu | 5.682200684399993 | 4.107928570570007 | 2.096321626389977 |
| Cu | 6.430814206615484 | 0.258344177296412 | 4.133869959603102 |
| Cu | 6.420451426427432 | 2.824016906415346 | 4.136678340747352 |
| Cu | 6.430897499969405 | -2.310623142422279 | 4.138666481563184 |
| Zn | 4.964047087723886 | 2.825141260004032 | 6.353207110439390 |

Cu111-Ag_H
56

| | | | |
|---|---|---|---|
| H | 1.772497625481297 | 3.930870466064846 | 8.431897017247174 |
| H | 1.726380724266561 | 5.246896891249410 | 9.915762258855738 |
| H | 2.537895091051429 | 3.321384978075937 | 9.722110367071279 |
| H | 2.550573326844790 | -0.948692120824669 | 9.883149649373669 |
| H | 3.748307550736158 | 1.590844640225358 | 9.916532979662703 |
| H | 3.579234068243166 | -0.037036979001874 | 8.345241810223024 |
| H | 4.614799307802402 | 2.909033073810739 | 9.957660816703267 |
| H | 4.600330718538308 | -0.594492181464564 | 9.412744205817617 |
| H | 6.059115419722285 | -1.487067683019525 | 8.274278443308384 |
| H | 6.283709139619936 | 3.384809245797862 | 8.453309953727603 |
| H | 6.242170060548975 | -3.141343895998602 | 9.498990192926746 |
| H | 0.230140546223615 | 2.927634045307156 | 9.469069096345313 |
| H | 0.869439904232226 | 6.733131510758717 | 9.959598158633726 |

| | | | |
|---|---|---|---|
| H | 3.161386863326332 | 2.814890892153971 | 7.191126172918432 |
| O | 1.701393113680834 | 3.869772466622044 | 9.421523415326341 |
| O | 1.731297854236718 | 6.246187015787258 | 10.267916141016958 |
| O | 3.736038136087851 | 2.535073148236895 | 10.213958112346239 |
| O | 3.713636319239231 | -0.125459889690233 | 9.330444151239007 |
| O | 6.073590484852041 | -1.457315524268461 | 9.276180024844233 |
| O | 6.267727957590989 | 3.555831015924179 | 9.437392417051448 |
| Cu | 0.494078305679993 | 0.256757570569980 | 0.000000000000000 |
| Cu | 0.494078305679993 | 2.824179229430008 | 0.000000000000000 |
| Cu | 0.494078305679993 | 5.391677911710007 | 0.000000000000000 |
| Cu | 0.550081742980313 | 5.355141115061846 | 6.208973327033660 |
| Cu | 0.545157264883521 | 0.263985059185532 | 6.189084548681303 |
| Cu | 0.493144766967783 | 2.809774607435440 | 6.168593876002674 |
| Cu | 1.235229116320017 | 1.540468399999967 | 2.096321626389977 |
| Cu | 1.235229116320017 | 4.107967082279965 | 2.096321626389977 |
| Cu | 1.235229116320017 | 6.675388741139993 | 2.096321626389977 |
| Cu | 1.986088228597327 | 2.812129491004262 | 4.129272994438948 |
| Cu | 1.990413024515931 | 0.263140316101479 | 4.142018732268095 |
| Cu | 1.989287187679711 | 5.370559405629442 | 4.141384138019083 |
| Cu | 2.717597441839991 | -1.026991770570019 | 0.000000000000000 |
| Cu | 2.717597441839991 | 1.540429888290010 | 0.000000000000000 |
| Cu | 2.717597441839991 | 4.107928570570007 | 0.000000000000000 |
| Cu | 2.680014579260822 | 1.487514488264612 | 6.239657810071907 |
| Cu | 2.728577430565735 | -1.045607461552222 | 6.189092615051075 |
| Cu | 2.686173034818657 | 4.121892324609107 | 6.211428771712170 |
| Cu | 3.458748252480015 | 0.256719058859968 | 2.096321626389977 |
| Cu | 3.458748252480015 | 2.824217741139966 | 2.096321626389977 |
| Cu | 3.458748252480015 | 5.391639399999995 | 2.096321626389977 |
| Cu | 4.201983451881743 | -1.035878949846011 | 4.126559955193639 |
| Cu | 4.202527413676891 | 4.104682028982385 | 4.126756426572383 |
| Cu | 4.202196976685009 | 1.525284595023384 | 4.132809358411998 |
| Cu | 4.941049873760016 | -2.310702600000033 | 0.000000000000000 |
| Cu | 4.941049873760016 | 0.256719058859995 | 0.000000000000000 |
| Cu | 4.941049873760016 | 2.824217741139993 | 0.000000000000000 |
| Cu | 4.953098155428109 | -2.277718456131136 | 6.175046271024421 |
| Cu | 4.960254523910526 | 0.180071682588140 | 6.183860288713289 |
| Cu | 5.682200684399993 | -1.026991770570018 | 2.096321626389977 |
| Cu | 5.682200684399993 | 1.540506911709979 | 2.096321626389977 |
| Cu | 5.682200684399993 | 4.107928570570007 | 2.096321626389977 |
| Cu | 6.426518318725216 | 0.261876484771479 | 4.143854031737616 |
| Cu | 6.425905139112688 | 2.814043235593710 | 4.140726996668192 |
| Cu | 6.427624373801635 | -2.328851887468155 | 4.139270422407569 |
| Ag | 4.982545874470552 | 2.798667963734576 | 6.450099002899911 |
